# The origins of climate‐diversity relationships and richness patterns in Chinese plants

**DOI:** 10.1002/ece3.9607

**Published:** 2022-12-12

**Authors:** Guilin Wu, John J. Wiens

**Affiliations:** ^1^ Hainan Jianfengling Forest Ecosystem National Field Science Observation and Research Station, Research Institute of Tropical Forestry Chinese Academy of Forestry Guangzhou Guangdong China; ^2^ Department of Ecology and Evolutionary Biology University of Arizona Tucson Arizona USA

**Keywords:** China, climate, diversification, niche conservatism, species richness, time‐for‐speciation

## Abstract

A major goal of ecology and evolutionary biology is to explain geographic patterns of species richness. Richness is often correlated with climatic variables. However, the processes underlying these climate‐diversity relationships remain poorly understood. Two potential hypotheses to explain these relationships involve: (i) faster diversification rates (speciation minus extinction) in high‐richness climates and (ii) earlier colonization of high‐richness climates, allowing more time for speciation to build up richness. Few studies have tested these hypotheses directly, and most focused on animal clades with limited richness. In this study, we test these hypotheses in Chinese angiosperms, encompassing ~10% of Earth's plant species, using large‐scale phylogenetic, climatic, and distributional data including 26,977 species. We find that climatic zones that were colonized earlier have higher species richness. By contrast, relationships between diversification rates and richness of climatic zones are often nonsignificant or negative. Our study reveals that even when richness is strongly correlated with climate, the underlying explanation may still be rooted in phylogenetic history. Thus, climate may not be a competing explanation for richness patterns relative to colonization times and diversification rates. We also show that the timing of colonization can be crucial for explaining richness patterns. Yet, many recent studies have ignored this explanation and instead have focused solely on rates of speciation and diversification as drivers of diversity gradients.

## Graphical abstract

Species richness is often strongly correlated with climatic variables, but what explains these strong climate‐richness relationships? Here, we address this question by analyzing climatic, distributional, and phylogenetic data from ~27,000 species of Chinese plants (~10% of the world's species). We find that climates with high species richness are explained by longer time in those climatic zones, not by faster rates of diversification.

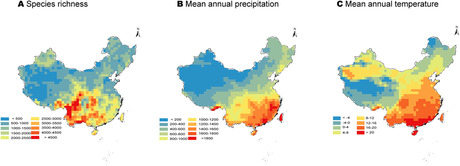

## 1 INTRODUCTION

Understanding the causes of species richness patterns is a long‐standing challenge at the intersection of ecology, evolutionary biology, and biogeography (Fine, 2015; Mittelbach et al., 2007; Pianka, 1966; Willig et al., 2003). Richness patterns are often correlated with climatic variables (Buckley & Jetz, 2007; Currie et al., 2004; Hawkins et al., 2003). Yet, the richness of climatic zones, habitats, and regions can only be directly changed by speciation, extinction, and dispersal (e.g., Ricklefs, 1987). Therefore, climate‐richness relationships should ultimately be explained by the effects of climate (either direct or indirect) on these three processes (e.g., Ricklefs, 2006; Wiens & Donoghue, 2004). However, few studies have tested how these processes interact with climate to generate climate‐richness relationships and spatial richness patterns (Kozak & Wiens, 2012; Lv et al., 2016; Wiens et al., 2011, 2013).

There are several hypotheses that can potentially explain the origins of climate‐richness relationships (e.g., energy‐richness, physiological tolerance, speciation rate; Currie et al., 2004). But only some hypotheses relate directly to speciation, extinction, and dispersal. Two broad hypotheses can explain climate‐richness relationships in terms of these processes (Ricklefs, 2006; Wiens, 2011). One is that certain climatic conditions promote faster diversification rates, where diversification is the rate of species accumulation over time (speciation minus extinction). Thus, if certain climatic conditions promote speciation and/or reduce extinction, then regions with those climates should have higher richness because the clades there proliferated more rapidly (all else being equal). The second hypothesis is that climates that presently have higher richness were colonized earlier by extant lineages than other climates. These climatic zones then have more time for richness to accumulate through speciation (time‐for‐speciation effect: Stephens & Wiens, 2003). This hypothesis assumes that most species occur under a limited range of climatic conditions and that dispersal among climatic regimes is limited (potentially manifested as phylogenetic conservatism in climate, with close relatives occurring in similar conditions). These two hypotheses (diversification rate and time) are not mutually exclusive. For example, habitats with more extinction (i.e., decreased diversification rates) may lose entire clades, leaving less time for speciation to build up richness after recolonization (Miller & Wiens, 2017). “Ecological limits” on richness have been considered an alternative to the time and diversification‐rate hypotheses (e.g., Mittelbach et al., 2007). However, ecological limits cannot directly change richness but can impact diversification rates and colonization times (Pontarp & Wiens, 2017). Therefore, ecological limits are not an alternative to the time and diversification‐rate hypotheses.

Relatively few studies have tested both the diversification‐rate and time hypotheses to explain climate‐richness relationships. A few studies have tested both hypotheses for climate‐richness relationships within some moderately sized families of vertebrates (~100–1000 species; hylid frogs: Wiens et al., 2011; plethodontid salamanders: Kozak & Wiens, 2012; phrynosomatid lizards: Wiens et al., 2013; cricetid rodents: Lv et al., 2016). These studies supported the hypothesis that colonization time (not diversification rates) explains these relationships. However, this hypothesis should be tested more broadly (e.g., in plants).

Here, we use Chinese angiosperms to test the causes of climate‐richness relationships at an unprecedented scale. Chinese angiosperms include ~28,000 species (Lu et al., 2018), encompassing ~10% of all land plants (The Plant List, 2013). Among these species, ~57% are endemic (15,960; Wang et al., 2015). Thus, most species originated in situ, especially since splits between endemic and non‐endemic sister species almost certainly occurred in China also. Thanks to work by Lu et al. (2018), new resources make Chinese angiosperms an excellent model system for addressing this question. These resources include: (a) time‐calibrated phylogenies incorporating most genera and species; (b) range maps for all species; and (c) climatic data (Lu et al., 2018). Using these (and similar) resources, there have now been important studies on the phylogenetic structure of Chinese plant assemblages (Gheyret et al., 2020; Lu et al., 2018; Qian et al., 2019). However, these studies did not test the causes of climate‐richness relationships. Similarly, Su et al. (2020) analyzed climate‐richness relationships among Asian plants but did not address whether colonization times or diversification rates explained these patterns.

In this paper, we test how richness patterns in Chinese angiosperms are related to climate, diversification rates, and colonization times. We first characterize the climatic distributions of Chinese angiosperm species. We divide the range of precipitation and temperature variables across China into bins (climatic zones defined by a range of values). We characterize the richness of these bins at the local‐assemblage scale (mean richness of grid cells within a given climatic bin) and regional scale (total richness of a climatic bin across China). We then test whether the richness of these climatic zones is explained by diversification rates or colonization times. Specifically, we estimate the diversification rate of each species and the mean rate for each climatic bin. We then test for relationships between richness and diversification rates of climatic bins. We also reconstruct ancestral climate niche values on the phylogeny, estimate the oldest colonization of each climatic bin, and test for relationships between richness and colonization times of climatic bins. Our results support the hypothesis that colonization times (not diversification rates) generally explain climate‐richness relationships in Chinese angiosperms.

## 2 MATERIALS AND METHODS

### 2.1 Phylogenetic and distributional data

The phylogenetic tree and distributional and climatic data were from Lu et al. (2018). We included 26,977 species and 2592 genera, including 96% of species and 90% of genera in China. We performed most analyses on a consensus of these 1000 trees (Data S1). We also performed separate analyses on five trees to address uncertainty in the phylogeny (Data S2–S6). These results were similar to those from the consensus tree.

The distribution data were from published national and provincial floras, and local floras, checklists, and herbarium records (Lu et al., 2018). Additional details about the phylogenetic, distributional, and climatic data are given in Methods S1.

### 2.2 Climate‐richness relationships

We characterized each grid cell based on its values for MAP (mean annual precipitation sum) and MAT (mean annual air temperature). These are two standard descriptors of large‐scale climate. Following standard practice in similar studies, we then estimated regional and local species richness for different large‐scale climatic zones by dividing the overall range of MAP and MAT values among grid cells into bins (*n* = 17 and 14, respectively). Details and further justification are given in Methods S1. Richness in each bin was based on the range of MAP and MAT values that each species occurred in. For example, if one species occurred in grid cells with MAP from 250–350 mm, it was included in the bins for 200–300 and 300–400. We refer to these as estimates of regional richness.

We also estimated the mean richness of grid cells within each bin. Specifically, we counted all grid cells with mean climatic values within the range for that bin. We then estimated the mean richness across these bins (this corrects for the different areas of climatic zones at regional scales). We refer to these estimates as mean “local” richness (although grid cells are much larger than local communities). Data on bin richness are in Data S7.

We estimated the relationship between richness and climatic variables using linear regression with *stats* in R version 1.1.456 (R Studio Team, 2016). We also used quadratic equations to fit this relationship (following Khine et al., 2019). We compared the relative fit of these linear and curvilinear models using the Akaike (1974) information criterion (AIC) for each model.

### 2.3 Diversification‐rate hypothesis

We tested whether climatic zones with higher richness have species that belong to clades with higher diversification rates. We estimated the mean diversification rate among species in each bin. We then tested for a relationship between richness and mean diversification rates across bins. We also tested for general relationships between climate and diversification rates of clades.

We used two general approaches to estimate diversification rates: genus level and species level (details in Methods S1). For the first approach, each species was assigned to a genus, and a diversification rate was estimated for each genus. Each species was then assigned the diversification rate of its genus. The mean diversification rate for a bin was the mean rate across all species in that bin. To test the relationship between diversification rates and richness across bins, we used linear regression in R. We did not account for phylogeny in these analyses because there is no phylogeny among bins.

The net diversification rate for each genus was estimated using the method‐of‐moments estimator for stem‐group ages (Magallón & Sanderson, 2001; MS estimator hereafter). This approach allows for thousands of different rates across the tree, and can give accurate rate estimates when rates vary strongly over time within clades (Meyer et al., 2018), between subclades within clades (Meyer & Wiens, 2018), and when rates are faster in younger clades and decoupled from richness (Kozak & Wiens, 2016). Thus, it does not require constant rates to be accurate. We used GEIGER version 2.0. (Harmon et al., 2008; Pennell et al., 2014) to estimate rates. Other details are in Methods S1.

Diversification rates for each genus were first calculated using only species in China. These rates should be most relevant for understanding Chinese richness patterns. Then, in separate analyses, the global richness of each genus was used. However, these two approaches gave similar results (see below).

The second general approach involved species‐level rates (i.e., each species with a different rate). We estimated the diversification‐rate statistic (DR) for each species as the inverse of its mean equal‐split measure (Jetz et al., 2012) using the R package PICANTE version 1.8 (Redding & Mooers, 2006). We generally used the mean value of rates among species but also included median rates. We also explored excluding a few species with exceptionally high rates (Methods S1; Figure S1).

We conducted these two analyses across all species, and within the most‐species‐rich families. We initially included the 50 richest families, but three contained only one genus in China (Aquifoliaceae, Balsaminaceae, Begoniaceae). This made it impossible to address variation in genus‐level rates in these families. We included 47 families, representing ~80% of the species in our tree (and in China).

We also tested for relationships between diversification rates of genera and their mean values of MAP and MAT (Data S8–S10). First, we estimated the mean MAP and MAT of each species. The mean MAP (and MAT) of each species was the mean value across all grid cells where it occurred. The mean MAP and MAT for each genus was the average of the mean values for all species in that genus. We also tested the relationships between diversification rates of families (using both MS and DR methods) and their mean values of MAP and MAT, as for genera.

We tested the relationship between diversification rates and climatic values of genera (2048) and families (235) using phylogenetic generalized least squares regression (PGLS: Martins & Hansen, 1997) with the R package *caper* version 0.5.2 (Orme, 2013). Details are in Methods S1. A potential weakness of this analysis is that it includes climatic data only for species in China. We therefore performed supplementary analyses in which we only included the 484 genera that occurred predominantly in China (≥60% of species occurring in China).

Diversification rates, clade ages, richness, and mean climatic values of genera and families are in Data S10, along with species‐level rate estimates.

### 2.4 Time hypothesis

To address the time hypothesis, we tested for a positive relationship between the richness of each MAP and MAT bin and the estimated time when that bin was first colonized (Data S7). To estimate the colonization time, we performed ancestral reconstructions of MAP and MAT. The full tree (26,977 species) was too large for the methods used (see below). We therefore performed ancestral reconstructions at the species level within each genus. We then performed reconstructions on the genus‐level tree (2048 genera), using the ancestral value for each genus as the data for each tip (genus). We also performed separate analyses within the 47 largest families, using values for each species (mean across grid cells). We obtained a species‐level tree within each family using the function drop. tip in the R package *ape* version 5.3 (Paradis & Schliep, 2019).

Reconstructions were primarily performed using the mvBM (multiple variance Brownian motion) model in the R package E_VOMAP_ version 0.0.0.9000 (Smaers & Mongle, 2019). Unlike a standard Brownian motion (BM) model, which assumes a single mean and variance for the rate across all branches, mvBM allows for different rates along different branches (Smaers et al., 2016; Smaers & Mongle, 2017). Simulations suggest that this approach is relatively accurate for reconstructing ancestral‐trait values (Smaers et al., 2016; Smaers & Mongle, 2017). Although this approach assumes a BM model, the high levels of phylogenetic signal found in both MAP and MAT are consistent with this model (see below).

We also evaluated the fit of these variables to four standard models: BM, estimated lambda (LA), Ornstein‐Uhlenbeck (OU; single peak), and white noise (WN). We used mean species values of genera for MAP and MAT, the genus‐level tree, and the function fitContinuous in the R package GEIGER, version 2.0 (Pennell et al., 2014). We compared the Akaike information criterion (AIC) of each model. The best‐fitting model (lowest AIC) was generally LA (Data S11), for the overall tree, the five trees, and 96% of the 50 family‐level trees. Importantly, LA is identical to BM when the phylogenetic signal is high. Nevertheless, we repeated the downstream analyses using LA and found similar results (Data S12). However, we preferred using mvBM because it allowed for rate heterogeneity.

Following standard approaches used in previous studies of climate‐richness relationships (e.g., Kozak & Wiens, 2012; Wiens et al., 2011, 2013), we identified the first colonization time based on the oldest node that was reconstructed as occurring in each MAP and MAT bin. For example, for the 300–400 mm bin of MAP, we found the oldest node that was reconstructed with any value in this range and then used the age of that node as the minimum time of the first colonization. In some cases, an extant species represented the oldest colonization of a bin. In these instances, we calculated the colonization time as half that species' age. Simulations suggest that these analyses are not necessarily biased to reconstruct climatic bins with the most species as the oldest (Kozak & Wiens, 2012; Wiens et al., 2013). These analyses should not require that we accurately estimate the precise timing of the first colonization of each climatic bin. Instead, we are testing whether lower richness habitats were generally colonized more recently than higher richness habitats.

To test for niche conservatism, we estimated phylogenetic signal (Pagel, 1999) in both MAP and MAT. High signal corresponds to covariation with the phylogeny, such that closely related species share similar trait values, indicating strong conservatism (Wiens et al., 2010). To estimate the signal, we used the function “phylosig” in the R package *phytools* version 0.6‐99 (Revell, 2012). We also tested whether the observed signal differed significantly from zero. We analyzed the genus‐level tree and the 50 largest families. Assessing signal is a conservative test of niche conservatism since traits can be conserved without showing significant signal (e.g., under an OU model of stabilizing selection; Revell et al., 2008; Wiens et al., 2010).

### 2.5 Patterns within families

We also examined whether time and/or diversification rates explained richness patterns within families, to evaluate the robustness of the results across Chinese angiosperms. We examined the 47 largest angiosperm families in China, which together include 77% of all species in China. Within each family, we estimated relationships between local and regional richness and diversification rates, and between richness and colonization times. Diversification rates were estimated based on stem‐group ages of genera with ε = 0.5. We tallied how often (i.e., how many families) there was a significant relationship (*p* < .05) between richness and diversification rates, colonization time, both, or neither (Table 1). We also focused on the 10 richest families in China, which encompass 42% of the species in the tree. Full results are given in Data S13–S14.

**TABLE 1:** Number of families in which species richness patterns were significantly related to colonization time, diversification rate, both or neither.

		MAP		MAT	
		Local	Regional	Local	Regional
47 families	Time not rate	20	13	9	7
	Rate not time	9	11	11	18
	Both time and rate	12	15	4	1
	Not time or rate	6	8	23	21
10 families	Time not rate	7	3	4	1
	Rate not time	1	1	2	4
	Both time and rate	2	4	0	0
	Not time or rate	0	2	4	5

*Note*: We present results for relationships between richness of climatic bins (local and regional) and diversification rates and colonization time for the 47 most‐species‐rich families in China (including 77% of the species in our tree). These are followed by results for the 10 richest families (including 42% of the species in our tree). Climatic variables are MAP (mean annual precipitation) and MAT (mean annual temperature). The mvBM model was used for ancestral reconstructions. Diversification rates were estimated based on stem‐group ages of genera with ε = 0.5, including only species in China. Use of alternative values of ε (0, 0.9) and global richness of genera yielded similar results (Data S13). Results for each family are given in Data S13.

Running many tests increases the potential for false positives. However, our goal here was to evaluate how often there was strong support for the diversification rate vs. time hypotheses. The potential for some low *p*‐values by chance should not bias our assessment of the frequency of support for one hypothesis over the other (i.e., we expect similar numbers of false positives).

## 3 RESULTS

### 3.1 Richness patterns

**FIGURE 1:**
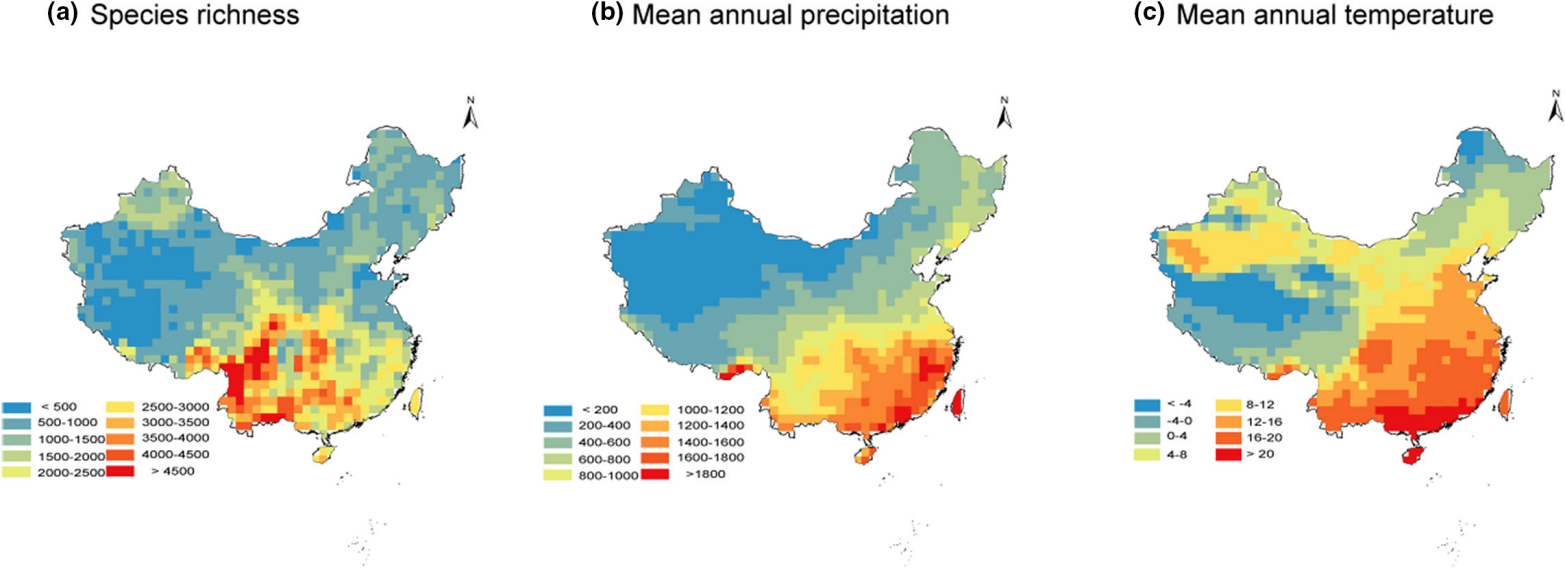
Patterns of species richness and climatic variation across China. Maps show 100 × 100 km grid cells, including (a) angiosperm species richness, (b) mean annual precipitation, and (c) mean annual temperature.

Spatial patterns of richness across China are summarized in Figure 1a, along with variation in precipitation and temperature (Figure 1b,c). Local species richness (100 × 100 km grid cells) is lowest in northern and western China overall, and highest in the mountainous southwestern provinces of Sichuan and Yunnan. Both temperature and precipitation are highest in southeastern China, and generally lower in northern and western China.

**FIGURE 2:**
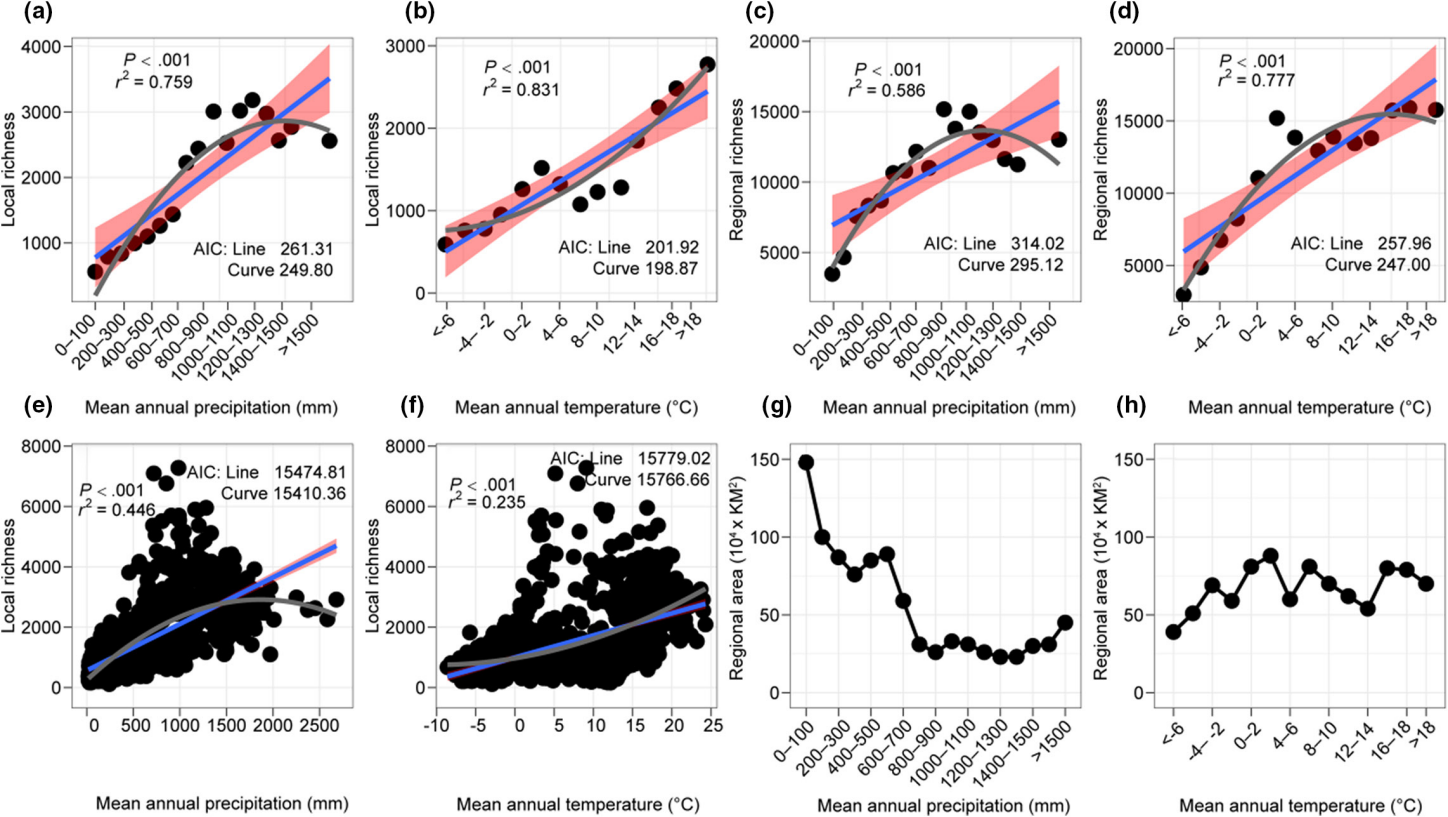
Patterns of species richness in Chinese angiosperms. Strong relationships between species richness of climatic zones and their mean value of MAP and MAT. Results are shown for mean local richness (across 100 × 100 km grid cells) across climatic zones for MAP (a) and MAT (b) and regional richness for MAP (c) and MAT (d). Relationships between local richness in each grid cell and grid‐cell values of MAP (e) and MAT (f). Linear and curvilinear relationships are shown in blue and gray, respectively. All curvilinear models had significantly better than linear models (Methods S1). Regional area of each climatic zone for MAP (g) and MAT (h). In a–f, the pink color indicates the 95% confidence interval for the linear regressions.

Local richness (Figure 2a,b) was highest in grid cells with intermediate values of MAP (800–1400 mm/year) and high values of MAT (>18°C). Regional richness (Figure 2c,d) was also highest at intermediate values of MAP (800–1400 mm/year) and higher values of MAT (2–18°C). There are strong, linear, positive relationships between the species richness of these climatic bins and their climatic values (Figure 2a–d), both at the local scale (MAP: *r* ^2^ = 0.759, *p* < .001; MAT: *r* ^2^ = 0.831, *p* < .001) and regional scale (MAP: *r* ^2^ = 0.586, *p* < .001; MAT: *r* ^2^ = 0.777, *p* < .001). Both MAP and MAT have significant, positive, linear relationships with local richness when including all grid cells (MAP: *r* ^2^ = 0.446, *p* < .001; Figure 2e; MAT: *r* ^2^ = 0.235, *p* < .001; Figure 2f). However, all relationships showed a better fit to curvilinear models (Methods S1). Based on the area of each bin (Figure 2g,h), much of the land‐surface area in China is relatively dry and cold.

We found a strong, positive relationship between local and regional richness for both MAP bins (*r* ^2^ = 0.804, *p* < .001; Figure S2a) and MAT bins (*r* ^2^ = 0.639, *p* < .001; Figure S2b). The relationship between regional area and regional richness was significantly negative among MAP bins (*r* ^2^ = 0.797, *p* < .001; Figure S2c) and significantly positive among MAT bins (*r* ^2^ = 0.452, *p* = .008; Figure S2d).

### 3.2 Diversification‐rate hypothesis

**FIGURE 3:**
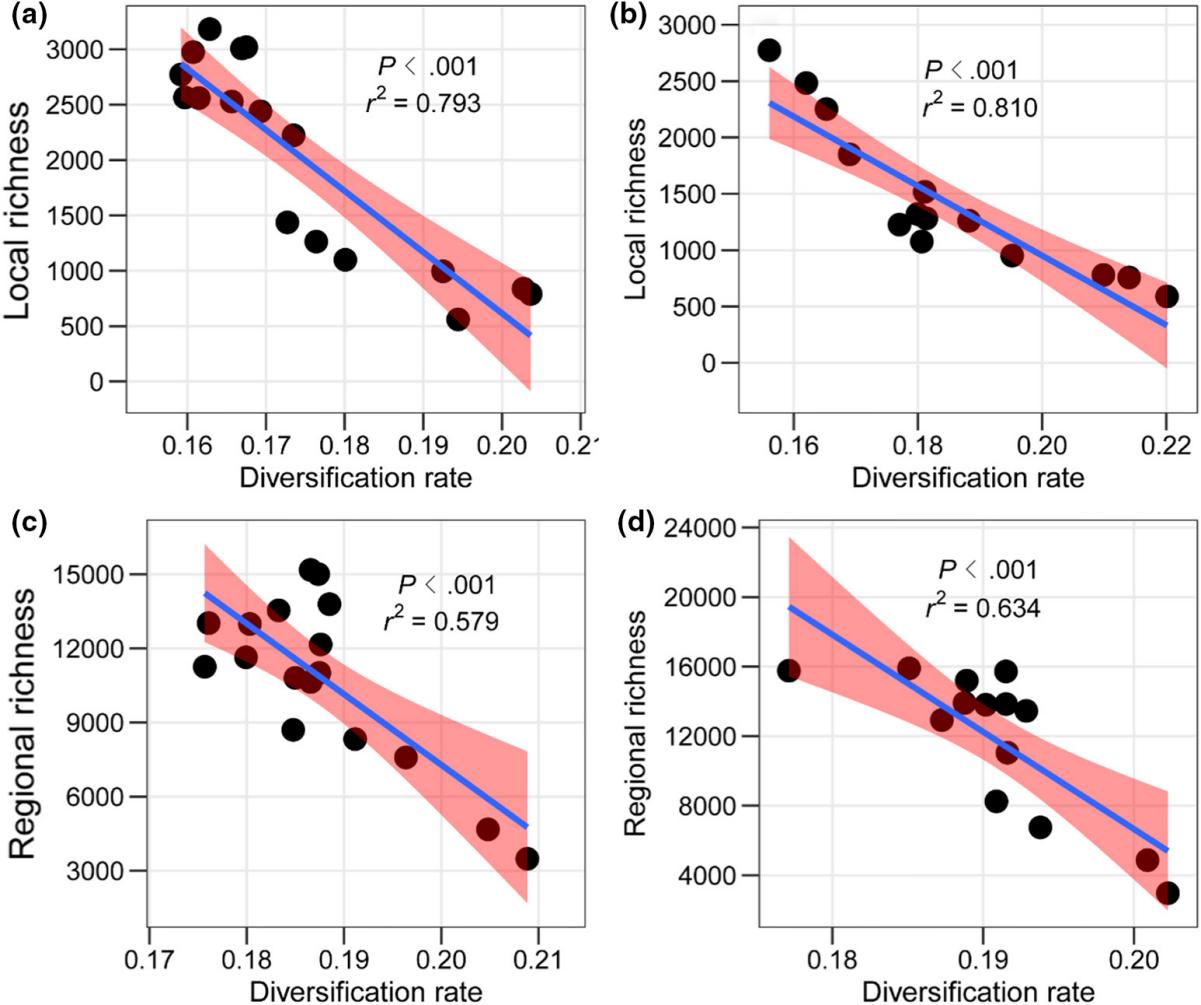
Significant negative relationship between mean diversification rates and species richness of climatic zones. Results are shown for mean local richness (across 100 × 100 km grid cells) across climatic zones for MAP (a) and MAT (b) and regional richness for MAP (c) and MAT (d). Diversification rates were estimated based on stem‐group ages of genera with ε = 0.5, including only species in China. Use of alternative values of ε (0, 0.9) and global richness of genera yields similar results (Table S1). Results using species‐level rates are more variable (Table S1). The pink color indicates the 95% confidence interval for regressions.

Using genus‐level diversification rates, we found strong, negative relationships between diversification rates and local richness of climatic zones, for both MAP and MAT (MAP: *r* ^2^ = 0.785–0.899, *p* < .001; MAT: *r* ^2^ = 0.794–0.957, *p* < .001; Figure 3a,b; Table S1). The range of *r* ^2^ values is for different relative extinction fractions (ε = 0, 0.5, and 0.9) used to estimate diversification rates (Methods S1). We also found strong negative relationships between genus‐level diversification rates and regional richness (MAP: *r* ^2^ = 0.457–0.802, *p* ≤ .003; MAT: *r* ^2^ = 0.616–0.896, *p* < .001; Figure 3c,d; Table S1). Similar patterns were also found in the five selected trees for both local and regional climatic zones (Table S2). Overall, these results strongly reject the diversification‐rate hypothesis (i.e., diversification rates should show a significant, positive relationship with richness to explain richness patterns).

Relationships between species‐level diversification rates (DR statistic) and richness were more variable (Table S1) but not consistently positive or significant. Relationships between mean rates and local richness were positive but weak (MAP: *r* ^2^ = 0.158, *p* = .115; MAT: *r* ^2^ = 0.263, *p* = .061). Results using median rates were significantly negative (MAP: *r* ^2^ = 0.277, *p* = .030; MAT: *r* ^2^ = 0.580, *p* = .002). By contrast, we found positive relationships between mean species‐level rates and regional richness (MAP: *r* ^2^ = 0.523 *p* < .001; MAT: *r* ^2^ = 0.723, *p* = .001). However, if species with the highest DR values (>1.5) were excluded (only 5% of species), relationships were not significant (MAP: *r* ^2^ = 0.052 *p* = .378; MAT: *r* ^2^ = 0.259, *p* = .063). Results using median DR values were similar for MAP but strongly positive for MAT (MAP: *r* ^2^ = 0.139 *p* = .141; MAT: *r* ^2^ = 0.473, *p* = .007). Across 5 selected species‐level trees (including 26,977 species from the posterior distribution of 1000 trees), relationships between median DR values and richness were not consistently positive or significant (Table S2).

We found only weak or nonsignificant relationships between climatic variables (MAP, MAT) and diversification rates among genera and families using phylogenetic regression (Figure S3; Table S3). Rates were estimated at the genus and family levels and using the DR statistic to estimate mean and median values among species within genera and families. Relationships were consistently negative, suggesting faster diversification in colder and drier climates. This pattern is the opposite of that predicted if diversification rates explain higher richness in warmer and wetter climates.

If only the 483 genera (3752 species in total) with the most species (≥60%) occurring in China are included, the relationships between diversification rates and climate are consistently weak (*r* ^2^ < 0.01) and nonsignificant (*p* > .05) across analyses when using both the MS estimator and median DR statistic (Table S4).

### 3.3 Time hypothesis and niche conservatism

**FIGURE 4:**
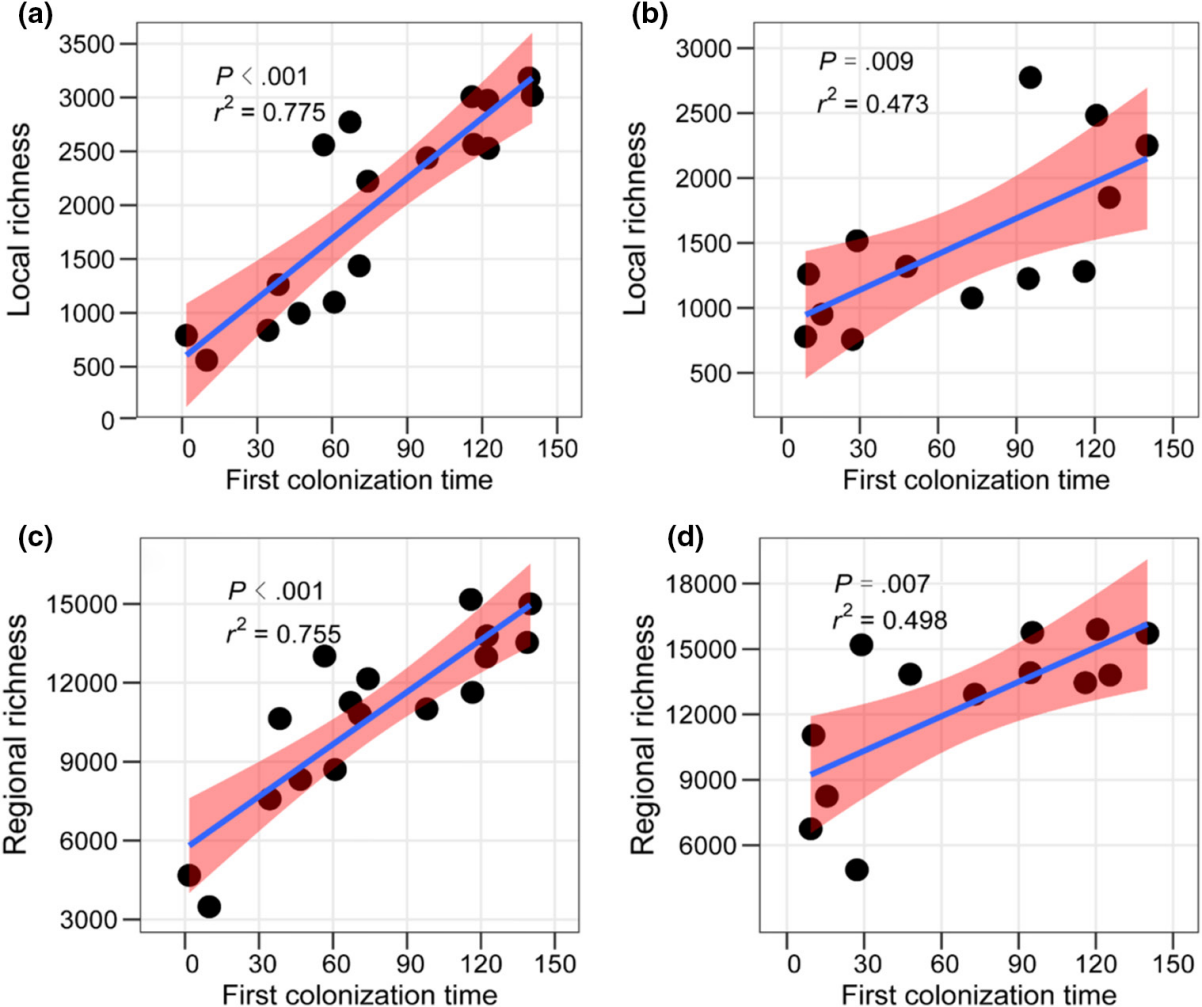
Strong relationships between species richness of climatic zones and their estimated time of first colonization. Results are shown for mean local richness (across 100 × 100 km grid cells) across climatic zones for MAP (a) and MAT (b) and regional richness for MAP (c) and MAT (d). Analyses are based on a genus‐level phylogeny and mean climatic values for genera. The pink color indicates the 95% confidence interval for regressions.

We found strong, positive relationships between the time of first colonization of each climatic zone and the local and regional species richness of these climatic zones, for both MAP (local: *r* ^2^ = 0.775; *p* < .001; regional: *r* ^2^ = 0.755; *p* < .001; Figure 4a,c) and MAT (local: *r* ^2^ = 0.473; *p* = .009; regional: *r* ^2^ = 0.498; *p* = .007; Figure 4b,d). The 5 selected trees gave similar results (Table S5). Results were also similar using the LA model instead of the mvBM model (Table S5).

The genus‐level tree showed significant phylogenetic conservatism for both climatic variables, with values of λ closer to the maximum of 1 than to 0, and with a likelihood‐ratio test significantly rejecting a λ of 0 (MAP: λ = 0.722; *P*_λ=0_ < 0.001; MAT: λ = 0.822; *P*_λ=0_ < 0.001).

### 3.4 Patterns within families

Our primary results were based on analyses across all families, but we also examined patterns within families. Within the 47 largest families in China, we estimated relationships between local and regional richness and diversification rates (MAP: Table S6; MAT: Table S7), and richness and colonization times (Tables S8–S9). Local richness of MAP bins (Table 1) was significantly related to colonization times (but not diversification rates) most frequently (43% of families), followed by both time and rates (26%), rates but not time (19%), and neither time nor rates (13%). The importance of time was even stronger among the 10 richest families in China, with richness patterns in 70% of families significantly explained by time alone, 20% by diversification rates and time, and 10% by rates and not time. Regional richness of MAP bins tended to be explained by both rates and time (Table 1), both among the 47 largest families (32%) and the 10 largest families (40%). But time alone tended to explain richness patterns more often than diversification rates alone (47 families: 28% vs. 23%: 10 families: 30% vs. 10%). These diversification rates were for stem‐group ages of genera, ε = 0.5, including only species in China.

For MAT, patterns within families were often not significantly explained by rates or by time (Table 1). Local richness tended to be explained by time alone or rates alone, whereas regional richness was more often related to diversification rates alone and not time. Results were similar using the LA model (Table S10).

The use of alternative values of ε (0, 0.9) and global richness of genera yielded similar results for both MAP and MAT (Data S13). Finally, among the 50 largest families, 41 had significant phylogenetic signal in MAP, and 46 had significant phylogenetic signals in MAT (Table S11). In summary, results within families were largely concordant with the between‐family results for MAP but were often more discordant within families, especially for MAT (and within‐family richness patterns were not necessarily identical to the larger between‐family patterns).

## 4 DISCUSSION

### 4.1 Overview

Understanding the causes of richness patterns is a fundamental goal in ecology, biogeography, and evolutionary biology. Using large‐scale, phylogeny‐based analyses encompassing ~27,000 species of Chinese angiosperms, we show that their climate‐richness relationships are explained primarily by colonization times of different climatic regimes, and not faster diversification in species‐rich climates. Specifically, our results suggest that regions with warmer, wetter climates in China have high richness today because these climates were colonized earlier than low‐richness climates (cooler, drier regions), allowing more time for richness to accumulate through speciation in these warmer, wetter climates. Thus, even strong climate‐richness relationships can be explained by the evolutionary and ecological processes that directly change species numbers: speciation, extinction, and dispersal.

### 4.2 Comparison to previous studies

Surprisingly, studies that have tested colonization time as an explanation for richness patterns remain uncommon. A recent systematic review (Li & Wiens, 2019) found few studies that tested this hypothesis. For example, recent large‐scale analyses of the latitudinal richness gradient have focused instead on speciation rates, including birds (e.g., Jetz et al., 2012), fish (Rabosky et al., 2018), and angiosperms (Igea & Tanentzap, 2020). These studies did not find positive relationships between richness and speciation rates. They did not test time as an alternative explanation and did not strongly support any explanation for richness patterns in these groups.

By contrast, previous analyses of climate‐richness relationships have supported colonization time as the primary cause of these patterns (in vertebrates; Kozak & Wiens, 2012; Lv et al., 2016; Wiens et al., 2013). Moreover, a recent survey found that time was generally important for explaining regional richness patterns in plants and animals (Li & Wiens, 2019), more often than area, diversification rates, or the frequency of dispersal.

Our results here (and these previous studies) imply that colonization time may be crucial for explaining climate‐richness relationships and richness patterns in general. However, the importance of colonization time may depend on the timescale. Diversification rates seem to be more important for explaining latitudinal richness patterns over deeper timescales (Schluter, 2016). This idea is also supported by simulations (Pontarp & Wiens, 2017). Importantly, angiosperms are relatively young, with a crown age < 150 Myr old in many recent estimates (e.g., Lu et al., 2018). Furthermore, diversification rates were significantly related to richness patterns within some angiosperm families (Table 1). Interestingly, in our study, diversification rates were not more important in older clades (Figures S4,S5). One potential explanation is rapid diversification in some clades in certain regions (e.g., Hengduan Mountains; Xing & Ree, 2017).

Our results are broadly consistent with other recent studies on Chinese angiosperms, although none addressed the specific questions addressed here. For example, grid cells with higher MAP and MAT have significantly older genera of Chinese angiosperms (Lu et al., 2018). This is potentially consistent with older colonization times, but the ages of named taxa may be unrelated to colonization times (Wiens, 2011). A study of phylogenetic structure and climate among Chinese angiosperms (Qian et al., 2019) found that regions with higher temperatures and precipitation have higher phylogenetic diversity and lower net relatedness. These patterns are potentially consistent with older colonization times of warmer, wetter climates. A recent study examined woody plant richness in east Asia and found that families with stronger climatic niche conservatism had stronger climate‐richness relationships (Su et al., 2020). This pattern is also consistent with the idea that higher richness in certain climates is caused by the combination of time and niche conservatism. In summary, none of these studies tested the hypotheses tested here, but all three are potentially consistent with the idea that colonization time drives climate‐richness relationships in Chinese angiosperms.

### 4.3 Potential sources of error

There are many potential sources of error that might impact our results. We address these at length in the final section of the Methods S1. These include changing climate over time (and changing area of climatic regimes) and topological uncertainty. These seem unlikely to overturn our conclusions. Most importantly, we included only Chinese angiosperms (~10% of Earth's plant species), which are largely endemic but not monophyletic. We addressed this issue in numerous ways, including restricting some diversification analyses to predominantly Chinese genera and including all species globally in others. None of these alternative analyses overturned our main conclusions. Similarly, for colonization time, it is unclear how species from other regions would overturn our conclusions about richness patterns within China (see Methods S1).

### 4.4 Areas for future research

Our results suggest several areas for future research. Future studies should address the impact of nonclimatic factors on richness patterns, including the high diversity of montane southwestern China and the role of elevational gradients (Rana et al., 2019; Sun et al., 2007). It would also be interesting to test these patterns at smaller spatial scales (e.g., 1 km grid cells). The effects of time on regional climate‐richness relationships can potentially help explain richness patterns at smaller scales (Kozak & Wiens, 2012; Wiens et al., 2011).

Another question is about the ecological and evolutionary processes that underlie the effects of colonization time. The ancestral environment for angiosperms overall was likely tropical (e.g., Kerkhoff et al., 2014; Zanne et al., 2014). More work is needed to identify the traits shared by lineages that colonized cooler and drier regions, and why these attributes are lacking in clades that remained in warmer and wetter climates. Our results show strong conservatism in these climatic variables, but more work will be needed to understand the processes underlying this pattern. These processes potentially include competition, limited genetic variation, selection, gene flow, pleiotropy, and trade‐offs among traits (Crisp & Cook, 2012; Wiens et al., 2010).

### 4.5 Species‐level diversification rates and richness patterns

Our results highlight the need for caution in interpreting the results of the DR statistic (Jetz et al., 2012) for explaining richness patterns. In some analyses, we found significant, positive relationships between diversification rates and richness that were strongly discordant with our other results. This pattern occurred because of exceptionally high rate estimates in ~5% of the species. The problem is not that these rate estimates are necessarily incorrect: the species involved appear to be very young, which is consistent with high rates. Instead, the problem is that the overall richness of a given region or climatic zone cannot be explained by very high rates in a tiny proportion of extant species. Other researchers should also be mindful of this issue.

## 5 CONCLUSIONS

Overall, using phylogenetic, climatic, and distributional data from ~27,000 species of Chinese angiosperms, we show that climate‐richness relationships are largely explained by colonization times and not diversification rates. Our results highlight the idea that strong relationships between climate and richness can be rooted in phylogenetic history (i.e., time‐for‐speciation effect) and that these climate‐richness relationships do not make evolutionary and biogeographic processes unimportant for explaining richness patterns. These results for Chinese angiosperms are consistent with those from vertebrate clades, and support the general importance of colonization time as a key factor for explaining richness patterns and climate‐richness relationships.

## AUTHOR CONTRIBUTIONS

**Guilin Wu:** Data curation (lead); formal analysis (lead); investigation (lead); methodology (lead); project administration (lead); visualization (lead); writing – original draft (equal); writing – review and editing (equal). **John J. Wiens:** Conceptualization (lead); writing – original draft (equal); writing – review and editing (equal).

## CONFLICT OF INTEREST

The authors declare that they have no conflicts of interest.

## Supporting information


**Appendix S1.** Supporting information

## Data Availability

Data availability statement The data that support the findings of this study are available as Supplementary Information, including Data S1–S15. These datafiles are also available on Dryad (https://doi.org/10.5061/dryad.tb2rbp048).
