# Supplementary material for: The origins of climate‐diversity relationships and richness patterns in Chinese plants
Source: Ecol Evol. 2022 Dec 12;12(12):e9607. doi: 10.1002/ece3.9607 (PMC9745389; doi:10.1002/ece3.9607)
Supplement: Supplementary file 1 — Appendix S1. Supporting information [file ECE3-12-e9607-s001.zip › ECE3_9607_Supporting_Information_27Sept2022.docx]

**Supporting Information**

Article title: Evolutionary and ecological drivers of species richness patterns in Chinese plants

This file includes

Supplementary Methods S1

Supplementary References

Tables S1–S11.

Figures S1–S6.

Other materials (in separate files)

Datafiles S1–S15

**Supplementary Methods S1**

**Phylogenetic tree and distributional data**

The phylogenetic tree and distributional data were from Lu et al. (2018)*.* That study used data from four plastid genes (*atpB, matK, ndhF,* *rbcL*) and one mitochondrial gene (*matR*). This initial dataset included 5,864 angiosperm species native to China, including 2,665 genera, representing ~92% of Chinese genera (Lu et al*.* 2018). With this dated genus-level chronogram as the backbone, these authors generated a species-level tree including 28,076 Chinese angiosperm species, encompassing nearly all Chinese species. After excluding 1,098 aquatic species (73 genera), the tree included 26,977 species, ~96% of all native, Chinese species. Unsampled species were randomly inserted into the genus-level tree using the R package S.PhyloMaker (Qian and Jin, 2016). Species were added to genera assuming that genera were monophyletic. However, for non-monophyletic genera (e.g. genus A paraphyletic with respect to B), unsampled species were assigned to the node of the most recent common ancestor of the relevant genera (e.g. A and B). All polytomies were then randomly resolved using MrBayes version 3.2 (Ronquist et al. 2012) and a birth-death model (Kuhn et al. 2011). A distribution of 1000 post-burn-in trees was generated, using topological and node-height constraints from the genus-level tree. One species (*Torricellia angulata*) was not included in the tree. We included 26,977 species and 2,592 genera, including 96% of species and 90% of genera in China.

We performed most analyses on a consensus of these 1000 trees. We used a maximum-clade credibility tree based on the mean heights of these trees using TREEANNOTATOR version 1.10 (Bouckaert et al. 2014). The tree was fully resolved (no polytomies). However, 892 (34%) of the 2,592 genera were non-monophyletic. The species-level tree is provided in Data S1. We also performed analyses on a subset of the trees to address the robustness of the results to uncertainty in the phylogeny (the 200th, 400th, 600th, 800th, and 1000th trees). These trees are given in Data S2–S6. Results based on these five trees were generally similar to those from the consensus tree, and therefore we did not explore a larger sample of trees.

Distributional and climatic data were generated for these same 26,977 species (Lu et al. 2018). China was divided into 100x100 km grid cells. A list of angiosperm species for each grid cell was compiled. Grid cells on the border or coast were excluded if <50% of the cell’s area included land in China. A total of 943 grid cells were included. Climatic data were downloaded from WorldClim 1.4 (<http://www.worldclim.org/>), with a spatial resolution of 10 min (~340 km^2^). These distributional data from Lu et al. (2018) included species composition, species richness, mean annual precipitation (MAP), and mean annual temperature (MAT) for each grid cell. Climate and richness data for each grid cell are given here in Data S10.

**Climate-richness relationships**

We characterized each grid cell based on its values for MAP and MAT, and then estimated regional and local species richness for different climatic regimes. To do this, we followed standard practice in previous studies on climate-richness relationships and divided the range of climatic values into a limited number of bins of equal size (i.e. same breadth of values; e.g. Wiens et al. 2011; Kozak and Wiens 2012; Wiens et al. 2013). Note that if we used raw values instead of bins, this would be similar to treating each grid cell as a separate data point, and would lead to problems of spatial autocorrelation. It would also prevent us from analyzing large-scale regional patterns.

Based on the range of mean MAP values (17.46–2679.93 mm/year) across grid cells in China, we initially divided the range of MAP values into 27 bins of 100 mm width, from 0–100 to 2600–2700. However, bins from 1600–2700 had few grid cells (seven bins with 0 or 1, mean=4.1, range=0–16). In contrast, the mean number of grid cells across the other bins was 56 (range=23–148). Therefore, we combined all grid cells >1600 mm into a single bin, yielding 17 bins in total.

Based on the range of MAT across grid cells (-8.6–24.3°C), we divided MAT into 16 bins, each of 2°C width (except the coldest and hottest, -8.6 to -6 and 22–24.3). There were only 37, 22, and 11 grid cells in the bins of 18–20, 20–22, 22–24.3°C, in contrast to the other bins (mean=67, range=39–88). We also combined these three bins into one bin, yielding 14 bins in total.

These bin widths and numbers were somewhat arbitrary. However, the number of bins was sufficient to obtain significant results. Furthermore, subdividing the range into more bins would yield greater statistical power, but many bins would be potentially redundant.

Richness in each bin was based on the range of MAP and MAT values that each species occurred in, based on their distribution among grid cells. For example, if one species occurred in grid cells with MAP from 250–350 mm/year, it was included in the bins for 200–300 and 300–400. We refer to these as estimates of regional richness.

We estimated the area of each bin by counting the number of equal-area grid cells encompassed in that bin. We then tested for a relationship between the richness of each bin and its area across China.

We also estimated the mean richness of grid cells within each bin. Specifically, we counted all grid cells with mean climatic values within the range for that bin. We then estimated the mean richness across these bins. We refer to these estimates as mean local richness, to contrast with regional richness (but acknowledging that these are not local communities). This approach corrects for differences in the total area associated with each bin when estimating regional richness. Data on richness of bins are given in Data S7.

We note that it would be hypothetically possible to include many other climatic variables besides MAT and MAP. However, the two variables used are standard descriptors of climate. For example, MAT helps characterize regions as tropical vs. temperate, whereas MAP helps distinguish regions that are arid vs. mesic. This is not necessarily true for variables based on annual variability in temperature or precipitation, or variables based on temperature and precipitation during shorter parts of the year (e.g. months or quarters).

It would also be possible to combine MAT and MAP into a single variable. However, we do not expect this to yield new insights, because these variables are already strongly related (*r*^2^=0.50; *P*<0.001) across all 943 grid cells (Fig. S6). Furthermore, we found broadly concordant results between these two variables separately.

We tested whether the regressions between richness and climate showed better fit to quadratic (curvilinear) models than linear models. We found that curvilinear relationships had lower AIC at the local scale (MAP: linear=261.31; curvilinear=249.80; MAT: linear=201.92; curvilinear=198.87; Fig. 2A,B) and regional scale (MAP: linear=314.02; curvilinear=295.12; MAT: linear=257.96; curvilinear=247.00; Fig. 2C,D), and based on grid cells (MAP: linear=15474.81 vs. curvilinear=15410.36; MAT: linear=15779.02 vs. curvilinear=15766.66; Fig. 2E,F). The comparisons between linear and curvilinear models were determined by using the function “anova” in the package *stats* in R version 1.1.456 (R Studio Team, 2016). All curvilinear models had significantly better fit than linear models (*P*<0.001).

**Diversification-rate hypothesis**

We tested whether climatic zones with higher richness have species that belong to clades with higher diversification rates. We estimated the mean diversification rate among the species in each bin. We then tested for a relationship between richness and mean diversification rates across bins. We also tested for general relationships between climate and diversification rates of clades.

We used two general approaches to estimate diversification rates: genus-level and species-level. First, each species was assigned to a genus, and a diversification rate was estimated for each genus. Each species was then assigned the diversification rate of its genus. The mean diversification rate for a bin was the mean rate across all species in that bin. To test the relationship between diversification rates and richness across bins, we used linear regression in R version 1.1.456 (R Studio Team 2016). We also used R to estimate 95% confidence intervals for these relationships. We did not account for phylogeny in these analyses, because there is no phylogeny among bins.

The net diversification rate for each genus was estimated using the method-of-moments estimator for stem-group ages (Magallón and Sanderson 2001), referred to as the MS estimator hereafter. Based on simulations, this method can accurately estimate diversification rates under many conditions, including when rates are faster in younger clades (Kozak and Wiens 2016) and when rates vary within clades over time and between subclades (Meyer et al*.* 2018; Meyer and Wiens 2018). Therefore, this method does not require constant rates within or between clades to be accurate. Indeed, using this method allowed each genus to have a separate, independent estimate of diversification rate (allowing thousands of different rates across the genus-level tree). In contrast, the method BAMM (Rabosky 2014) assigns a limited number of rates to all clades across large trees (2–3), thereby assuming widespread rate homogeneity, even when the true diversification rates vary extensively among clades (Meyer and Wiens 2018). This pattern of underestimating rate variation was documented by the developer of BAMM in the paper in which it was first proposed (Rabosky 2014).

A recent paper suggested that the MS estimators are problematic (Rabosky and Benson 2021), but the justification for this claim is questionable at best. Strangely, that paper did not address whether or not these estimators accurately estimate diversification rates (as done by Kozak and Wiens 2016; Meyer and Wiens 2018; Meyer et al. 2018). Indeed, estimating diversification rates is what these methods are designed to do and the purpose that they are used for here. Instead, that paper (Rabosky and Benson 2021) applied the MS estimators to predict species richness patterns over time in the fossil record, which no previous studies have used these methods to do. This criticism is especially questionable because simulations have shown that the MS estimators can be accurate even when species richness and diversification rates are uncoupled (Kozak and Wiens 2016).

The MS estimator requires the richness and age for each genus. Furthermore, it also uses the relative extinction fraction (ε; extinction/speciation) to correct rate estimates for clades that are entirely unsampled due to extinction. This value is assumed across the entire tree rather than estimated for individual clades, and does not assume that all clades have the same extinction rate. Indeed, simulations show that the method can accurately estimates diversification rates when a single ε value is used across clades with very different extinction rates (Meyer and Wiens 2018; Meyer et al. 2018). Following standard practice, we used three values: two extreme (ε=0 and 0.9) and one intermediate (ε=0.5). However, different values had little impact on the results, and we primarily focused on the intermediate value. We obtained stem ages of genera from the species-level consensus tree and the five trees, then used GEIGER version 2.0. (Pennell et al. 2014) to estimate rates.

The stem-group estimator was used because, unlike the crown-group estimator, it is not affected by incomplete species sampling within clades (Meyer and Wiens 2018). Furthermore, even with complete sampling, the stem-group estimator is generally more accurate (Meyer and Wiens 2018). Finally, the stem-group estimator can be applied to genera with single species, whereas the crown-group age for monotypic genera is undefined.

Estimating these diversification rates was complicated by the non-monophyly of several genera. When a genus was non-monophyletic, we combined it with other genera to form the smallest monophyletic group possible (e.g. if genus A was paraphyletic with respect to B, we used a clade including only A and B). For ages, we used the stem age of the combined clade (e.g. stem age of A+B). Richness was the richness of the combined genera. The 892 non-monophyletic genera were combined to yield 348 clades that amalgamated two or more genera (Data S8). Thus, there were 2,048 genus-level clades, including 1,700 monophyletic genera and 348 combined clades. The 2,048 genus-level tree (trimmed from the consensus tree of 26,977 species) is given in Data S9. Richness, ages, and rates for all genera and clades are in Data S10.

Diversification rates for each genus were first calculated using only species in China. These rates may be the most relevant for understanding Chinese richness patterns. Then, in a separate set of analyses, the total global species richness of each genus was used. However, these two approaches gave similar results (see below). The species richness of each genus in China was based on the Flora of China (Wu et al. 2013).

To estimate the total global species richness of each genus, we used the R package TAXONLOOKUP version 1.1.5 (Pennell et al. 2016). The data source used for this package was The Plant List (2013) version 1.1. However, some endemic Chinese species were included in the Flora of China (Wu et al. 2013) but were not accepted by The Plant List. Therefore, we also included “unresolved” species (i.e. not yet assigned a status of “Accepted” or “Synonym”). We used both accepted and unresolved species to estimate the global species richness of each genus. For more details see Data S10.

The second general approach involved directly estimating diversification rates at the species level, allowing each species to have a different rate. We estimated the diversification-rate statistic (DR) for each species in the tree as the inverse of its mean equal-split measure (Jetz et al*.* 2012) using the R package PICANTE version 1.8 (Redding and Mooers 2006). We generally used the mean value of rates among species at the local and regional scales, but we also included median rates. We note that simulations show that this method is accurate overall, but performs better at estimating speciation rates than net diversification rates (Title and Rabosky 2019). If certain climatic conditions enhance species richness by increasing diversification rates, then we assume that this will be reflected in both speciation and net diversification rates. We note that this method was found to be especially useful when rates vary among small groups of closely related species (Title and Rabosky 2019).

DR values ranged from 0.007–50.998 (Fig. S1). DR values seemed very high (>1.5 species/Myr) in 1406 species (5.2% of 26,977). These species had branch lengths from ~4,000–20,000 years. We also analyzed relationships between species richness and DR after removing these species. These high-rate species were distributed across most MAP and MAT bins (Fig. S1).

We conducted these two diversification analyses across all species simultaneously, and separately within the most species-rich families. We initially included the 50 richest families, but three contained only one genus in China (Aquifoliaceae, Balsaminaceae, Begoniaceae). This made it impossible to address how variation in genus-level diversification rates explained richness patterns within these three families. Therefore, we included 47 families, which represented ~80% of the species in our tree.

We also tested for a relationship between diversification rates of genera and their mean values of MAP and of MAT. First, we estimated the mean MAP and MAT of each species. The mean MAP (and MAT) of each species was the mean value across all grid cells in which the species occurred. The mean MAP and MAT for each genus was the average of the mean values for all species in that genus. We also tested the relationships between diversification rates of families (using both MS and DR methods) and their mean values of MAP and MAT, as described for genera.

We tested the relationship between diversification rates and climatic values of genera (2,048) and families (235) using phylogenetic generalized least squares regression (PGLS: Martins and Hansen 1997) with the R package *caper* version 0.5.2 (Orme 2013). For this analysis, we created a genus-level tree and family-level tree, pruning the chronogram to include a single, arbitrary species from each genus and family (the time-span to the root is the same for all species). Branch lengths were optimized using the maximum likelihood transformation (“lambda=ML”). Estimated values of lambda (Pagel 1999) were used, and kappa and delta were fixed at 1. A potential weakness of this analysis is that it includes climatic data only for species in China. We therefore performed supplementary analyses in which we only included the 484 genera that occurred predominantly in China (>60% of species occurring in China). We did not do this at the family level because relatively few of the most species-rich families in China occurred mostly in China (none of the 50 richest families have >30% of their global richness in China).

Diversification rates, clade ages, species richness, and mean climatic values of genera and families are given in Data S10, along with species-level rate estimates.

**Potential sources of error**

We acknowledge that there are many important sources of error that might impact our results. However, we think that these would generally make it harder to find the significant relationships that we found.

First, our analyses are based primarily on species occurring in China, whereas most families and genera are more broadly distributed. How might this impact our results? Our goal is to explain richness patterns in China. Therefore, the diversification rates of species in China are the most relevant to explaining these patterns, not rates of confamilials or congeners in other regions. Moreover, even if members of a genus had much higher diversification rates in North America than in China (for example), it is unclear how those higher rates would be relevant to richness patterns within China. Our analyses also include estimated rates for individual species, which should be most relevant to explaining patterns within China. Furthermore, the majority of Chinese angiosperm species are endemic (Wang et al. 2015), suggesting that most species did actually arise in China. Thus, the split between each endemic species and its sister species occurred in China, meaning that both the endemic species (~57% of Chinese species) and their sister species originated in China. We also include rates estimated for genera, including both species in China (in some analyses) and total global richness (in others). We also conducted analyses on genera with most species occurring in China. All four analyses generally agreed that diversification rates do not explain richness patterns within China.

A similar argument applies to analyses of colonization time and species richness. If members of a genus occur in arid regions in China but mesic regions in Europe, it is unclear how the habitat in Europe will be relevant to explaining richness patterns among habitats within China. Occurrence of species outside China might be an additional source of noise in estimating the causes of climate-richness relationships in China, but it is unclear how excluding these extralimital species would create false but statistically significant patterns. It is also important to note that (at least for MAP) the strong effect of colonization time on climate-richness relationships was generally also supported within the most species-rich families (Table 1). Thus, we subdivided our data, looked at a more recent timescale (i.e. patterns within families are younger than those across the whole tree), and still supported our main conclusions. It seems especially unlikely that the replicated support for this pattern is an artifact, especially without some specific mechanism that would generate statistically significant but misleading results.

Hypothetically the problem of extralimital species might explain the lack of a consistent, positive, significant relationship between diversification rates and climate. But we do not know of a specific mechanism by which this would happen, and especially not one that would generate the strong, significant negative relationships between diversification rates and richness that we often observed. Furthermore, for the diversification analyses, we used many approaches (both including and excluding extralimital species) which generally yielded similar answers. We also performed analyses including only genera in which most species occurred in China. These genera also showed no significant, positive relationships between the climatic variables and diversification rates, strongly suggesting that these variables do not explain richness patterns.

Second, climate has doubtless changed in the region over the relevant timeframe for Chinese angiosperms (e.g. mostly Cenozoic), including the expansion of temperate regions, the expansion and contraction of mesic regions, and more recent glacial and interglacial periods (e.g. Sun and Wang 2005; Guo et al. 2008). Our results show that climatic-niche variables show strong phylogenetic signal among Chinese angiosperms. Thus, it seems that many species and clades have likely shifted their geographic ranges to track suitable climates as climates have changed over time, rather than adapting quickly to changing conditions in situ. Indeed, the tight association between particular climates and particular plant clades is often used to infer past climates (e.g. Sun and Wang 2005). Furthermore, if past climate change had erased any trace of the past climatic niches of species and clades, then why do we find strong phylogenetic signal among these species now? And why do we find strong relationships between inferred colonization times and richness among thousands of species? We do not know of any plausible explanation for how past climate change would generate statistically significant but misleading results for these tests. Finally, our analyses are focused on explaining the richness of different climatic zones, not of specific geographic locations (e.g. specific grid cells). Therefore, the fact that climate in specific geographic locations has changed over time is not problematic here.

Similarly, our results do not address the impacts of the changing area of different climatic regimes over time (e.g. Fine and Ree 2006; Jetz and Fine 2012; Fine 2015). For example, temperate regions are thought to have expanded to their present extent only ~30–40 million years ago, and this is thought to have contributed to their lower diversity relative to tropical regions (e.g. Wiens and Donoghue 2004; Fine and Ree 2006). However, some authors have suggested that much of China was arid during the Paleocene and Eocene (Sun and Wang 2005), and the large mesic areas in southern and eastern China may be more recent (Guo et al. 2008). Thus, it is not so clear that time-integrated area could explain richness patterns for both (or either) climatic variable. Furthermore, area can only increase species richness by acting on speciation, extinction, and dispersal (e.g. by increasing diversification rates in clades in regions or climates with larger area; Fine 2015). Our results do not support the idea that more species-rich climates have higher diversification rates. Furthermore, recent analyses suggest that colonization time is more important than area for explaining richness patterns within clades (Li and Wiens 2019), which is concordant with our results here.

Third, we note that our examination of climatic variables is not exhaustive. We used two standard measures of annual climatic temperatures and precipitation (MAT and MAP). It might be that other climatic variables are also helpful in explaining spatial richness patterns in Chinese angiosperms. However, our main goal was to understand the origins of climate-diversity relationships. That is, we want to know why climatic variables show strong relationships with species richness. The relationships between spatial grid cells and these climatic variables are not perfect (MAP: *r*^2^=0.446, *P*<0.001; Fig. 2E; MAT: *r*^2^=0.235, *P*<0.001; Fig. 2F). However, when these climatic variables are divided into bins of similar sizes, there are very strong relationships between the climate of bins and their richness, at both the local scale (MAP: *r*^2^=0.759, *P*<0.001; MAT: *r*^2^=0.831, *P*<0.001) and regional scale (MAP: *r*^2^=0.586, *P*<0.001; MAT: *r*^2^=0.777, *P*<0.001). These are the climate-diversity relationships that we want to explain, and they are clearly very strong.

Fourth, we note that there are many other potential sources of error in our ancestral reconstructions, besides changing climates over time. The climate data are relatively coarse scaled for each bin (100x100 km), and there could be considerable climatic variation within many bins (especially bins in montane, tropical regions). Climatic values for each bin are then averaged across the range of each species to generate a mean value for each species. These mean values are then reconstructed on the phylogeny, and not the full range of climatic values that each extant or ancestral species occupied. Thus, there are numerous sources of error, which would presumably make it more difficult to find significant patterns. Nevertheless, we found strong relationships between time and richness of climatic bins and significant phylogenetic signal in these climatic variables (both across families and within many families). These analyses should not require that we precisely estimate the timing of the first colonization of each climatic bin. Instead, we are testing whether lower richness climatic zones (e.g. cooler, drier) were generally colonized more recently than higher richness climatic zones (e.g. warmer, wetter). Our results within China (suggesting longer occupation of more mesic, tropical climates) are consistent with results of broader analyses across angiosperms, which have suggested tropical ancestry for the group (e.g. Kerkhoff et al. 2014; Zanne et al. 2014)*.* We also note that our conclusions are very similar to those of smaller-scaled studies that used more fine-scale climatic data (1x1 km) for each species (Wiens et al. 2011; Kozak and Wiens 2012; Wiens et al. 2013; note that the spatial scale of the climatic data was unclear in Lv et al. 2016). Therefore, there is no reason to think that our conclusions are an artifact of using more coarse-scaled climatic data.

Finally, we note that the overall tree of 26,977 species was fully resolved but utilized randomly resolved polytomies at the species level. However, the main analyses were repeated on multiple trees, and the results were generally insensitive to different resolutions of these polytomies (Tables S2–S5). Furthermore, the analyses of genus-level diversification rates used a fully resolved tree among genera, and so should be insensitive to resolutions of species-level tips. In our analyses of species-level rates, the species’ rates and their climatic distributions remain associated regardless of the resolution of the polytomies. Analyses within families might be the most sensitive to random resolutions of species-level polytomies, but these results were often significant and often concordant with the overall results, especially for MAP (Table 1).

**References (not cited in main text)**

Bouckaert, R. et al. 2014. BEAST 2: a software platform for Bayesian evolutionary analysis. –PLoS Comput. Biol. 10: e1003537.

Fine, P. V. A., & Ree, R. H. 2006. Evidence for a time-integrated species-area effect on the latitudinal gradient in tree diversity. –Am. Nat.168: 796–804

Guo, Z.T. et al. 2008. A major reorganization of Asian climate by the early Miocene. Clim. Past 4: 153–174.

Jetz, W., & Fine, P. V. A. 2012. Global gradients in vertebrate diversity predicted by historical area productivity dynamics and contemporary environment. –PLoS Biol. 10: e1001292.

Kuhn, T. S. et al. 2011. A simple polytomy resolver for dated phylogenies. –Methods Ecol. Evol. 2: 427–436.

Pennell, M. W. et al. 2016. A simple approach for maximizing the overlap of phylogenetic and comparative data. –Methods Ecol. Evol. 7: 751–758.

Qian, H. & Jin, Y. 2016. An updated megaphylogeny of plants, a tool for generating plant phylogenies and an analysis of phylogenetic community structure. –J. Plant Ecol. 9: 233–239.

Rabosky, D. L. & Benson, R. B. 2021. Ecological and biogeographic drivers of biodiversity cannot be resolved using clade age-richness data. –Nat. Commun. 12: 1–10.

Rabosky, D. L. 2014. Automatic detection of key innovations, rate shifts, and diversity-dependence on phylogenetic trees. –PLoS One 9: e89543

Ronquist, F. et al. 2012. MrBayes 3.2: efficient Bayesian phylogenetic inference and model choice across a large model space. –Syst. Biol. 61: 539–542.

Sun, X. & Wang, P. (2005). How old is the Asian monsoon system? – Palaeobotanical records from China. –Palaeogeogr. Palaeocl*.* 222: 181–222.

Title, P. O. & Rabosky, D. L. 2019. Tip rates, phylogenies, and diversification: what are we estimating, and how good are the estimates? –Methods Ecol. Evol. 10: 821–834.

Wu, Z. Y. et al. (eds) Flora of China, Vol. 1–25 (Science Press & Missouri Botanical Garden Press, 1994–2013).

**Table S1**  Relationships between diversification rates and species richness for the consensus species-level tree (including 26977 species). MS indicates that genus-level diversification rates were estimated using the MS estimator (Magallón and Sanderson 2001). For these estimates, clade ages were based on stem ages and species richness was based either on species occurring in China (China) or all species in the genus (Global). Three different values of ε were used (0, 0.5, and 0.90). DR indicates that diversification rates were estimated using the DR statistic (Jetz et al. 2012). The baseline analyses (DR mean) included all species and summarized their rates using mean values among species. DR<1.5 used mean values, but only including species with DR values <1.5. DR median indicates that median values among species were used, instead of the mean. Negative and positive relationships between richness and diversification rates are indicated by - and +. Significant results (*P*<0.05) are boldfaced.

| Species richness | Diversification rate | MAP bins | |  | MAT bins | |  |
| --- | --- | --- | --- | --- | --- | --- | --- |
|  |  | *P* | *r*^2^ |  | *P* | *r*^2^ |  |
| Local | MS: China (ε=0) | **<0.001** | **0.785** | - | **<0.001** | **0.816** | - |
| richness | MS: China (ε=0.5) | **<0.001** | **0.789** | - | **<0.001** | **0.812** | - |
|  | MS: China (ε=0.9) | **<0.001** | **0.814** | - | **<0.001** | **0.794** | - |
|  | MS: Global (ε=0) | **<0.001** | **0.884** | - | **<0.001** | **0.954** | - |
|  | MS: Global (ε=0.5) | **<0.001** | **0.889** | - | **<0.001** | **0.954** | - |
|  | MS: Global (ε=0.9) | **<0.001** | **0.899** | - | **<0.001** | **0.957** | - |
|  | DR mean | 0.115 | 0.158 | + | 0.061 | 0.263 | + |
|  | DR < 1.5 | **0.007** | **0.394** | - | **<0.001** | **0.797** | - |
|  | DR median | **0.030** | **0.277** | - | **0.002** | **0.580** | - |
| Regional | MS: China (ε=0) | **<0.001** | **0.609** | - | **<0.001** | **0.650** | - |
| richness | MS: China (ε=0.5) | **<0.001** | **0.577** | - | **<0.001** | **0.616** | - |
|  | MS: China (ε=0.9) | **0.003** | **0.457** | - | **<0.001** | **0.631** | - |
|  | MS: Global (ε=0) | **<0.001** | **0.784** | - | **<0.001** | **0.891** | - |
|  | MS: Global (ε=0.5) | **<0.001** | **0.795** | - | **<0.001** | **0.889** | - |
|  | MS: Global (ε=0.9) | **<0.001** | **0.802** | - | **<0.001** | **0.896** | - |
|  | DR mean | **0.001** | **0.523** | + | **<0.001** | **0.723** | + |
|  | DR < 1.5 | 0.378 | 0.052 | + | 0.063 | 0.259 | + |
|  | DR median | 0.141 | 0.139 | + | **0.007** | **0.473** | + |

**Table S2**  Relationships between diversification rates and species richness for five selected trees (including 26,977 species from the posterior distribution of 1,000 trees). Diversification rates were estimated based on stem-group ages of genera with ε=0.5 and median values of the DR statistic among species in each genus (Jetz et al*.* 2012), including only species in China. Negative and positive relationships between richness and diversification rates are indicated by - and +. Significant results (*P*<0.05) are boldfaced.

| Climate zone | Trees | China (ε=0.5) | | | | |  | DR median | | | | |  |
| --- | --- | --- | --- | --- | --- | --- | --- | --- | --- | --- | --- | --- | --- |
|  |  | Regional richness | |  | Local richness | |  | Regional richness | |  | Local richness | |  |
|  |  | *P* | *r*^2^ |  | *P* | *r*^2^ |  | *P* | *r*^2^ |  | *P* | *r*^2^ |  |
| MAP | 200th | **< 0.001** | **0.620** | - | **< 0.001** | **0.796** | - | 0.837 | 0.003 | - | **0.023** | **0.300** | - |
|  | 400th | **< 0.001** | **0.599** | - | **< 0.001** | **0.787** | - | 0.332 | 0.063 | - | **<0.001** | **0.689** | - |
|  | 600th | **< 0.001** | **0.622** | - | **< 0.001** | **0.809** | - | 0.437 | 0.041 | + | **0.027** | **0.285** | - |
|  | 800th | **< 0.001** | **0.624** | - | **< 0.001** | **0.806** | - | 0.416 | 0.045 | + | 0.064 | 0.210 | - |
|  | 1000th | **< 0.001** | **0.610** | - | **< 0.001** | **0.806** | - | 0.199 | 0.108 | + | 0.252 | 0.086 | - |
| MAT | 200th | **<0.001** | **0.679** | - | **<0.001** | **0.819** | - | 0.317 | 0.083 | + | **<0.001** | **0.709** | - |
|  | 400th | **<0.001** | **0.647** | - | **<0.001** | **0.807** | - | 0.772 | 0.007 | + | **<0.001** | **0.801** | - |
|  | 600th | **<0.001** | **0.688** | - | **<0.001** | **0.829** | - | 0.053 | 0.278 | + | **<0.001** | **0.675** | - |
|  | 800th | **<0.001** | **0.689** | - | **<0.001** | **0.826** | - | **0.031** | **0.333** | + | **0.002** | **0.559** | - |
|  | 1000th | **<0.001** | **0.671** | - | **<0.001** | **0.826** | - | **0.006** | **0.482** | + | **0.010** | **0.440** | - |

**Table S3**  Relationships between diversification rates of genera and their mean values of MAP and MAT based on phylogenetic regression. MS indicates that genus-level diversification rates were estimated using the MS estimator. For these estimates, clade ages were based on stem ages and species richness was based either on species occurring in China (China) or all species in the genus (Global). Three different values of ε were used (0, 0.5, and 0.90). DR indicates that diversification rates were estimated using the DR statistic. The baseline analyses (DR mean) included all species and summarized their rates using mean values among species. DR<1.5 used mean values, but only including species with DR values <1.5. DR median indicates that median values among species were used, instead of the mean. Negative and positive relationships between richness and diversification rates are indicated by - and +. Significant results (*P*<0.05) are boldfaced.

| Independent variable | Dependent variable | Genus level | |  |  | Family level | |  |
| --- | --- | --- | --- | --- | --- | --- | --- | --- |
|  |  | *P* | *r*^2^ |  |  | *P* | *r*^2^ |  |
| MAP | MS: China (ε=0) | **0.037** | **0.002** | **-** |  | **0.017** | **0.024** | **-** |
|  | MS: China (ε=0.5) | **0.046** | **0.002** | **-** |  | **0.008** | **0.030** | **-** |
|  | MS: China (ε=0.9) | 0.090 | 0.001 | **-** |  | **0.001** | **0.044** | **-** |
|  | MS: Global (ε=0) | 0.145 | 0.001 | **-** |  | 0.405 | 0.003 | **-** |
|  | MS: Global (ε=0.5) | 0.145 | 0.001 | **-** |  | 0.344 | 0.004 | **-** |
|  | MS: Global (ε=0.9) | 0.139 | 0.001 | **-** |  | 0.181 | 0.008 | **-** |
|  | DR mean | 0.081 | 0.041 | **-** |  | 0.192 | 0.007 | **-** |
|  | DR < 1.5 | **<0.001** | **0.006** | **-** |  | **0.031** | **0.020** | **-** |
|  | DR median | **0.023** | **0.003** | **-** |  | 0.185 | 0.008 | **-** |
| MAT | MS: China (ε=0) | **0.012** | **0.003** | **-** |  | **<0.001** | **0.057** | **-** |
|  | MS: China (ε=0.5) | **0.012** | **0.003** | **-** |  | **<0.001** | **0.065** | **-** |
|  | MS: China (ε=0.9) | **0.018** | **0.003** | **-** |  | **<0.001** | **0.082** | **-** |
|  | MS: Global (ε=0) | 0.141 | 0.001 | **-** |  | 0.087 | 0.013 | **-** |
|  | MS: Global (ε=0.5) | 0.136 | 0.001 | **-** |  | 0.066 | 0.014 | **-** |
|  | MS: Global (ε=0.9) | 0.105 | 0.001 | **-** |  | 0.024 | 0.022 | **-** |
|  | DR mean | **0.013** | **0.003** | **-** |  | **0.038** | **0.018** | **-** |
|  | DR < 1.5 | **<0.001** | **0.018** | **-** |  | **<0.001** | **0.047** | **-** |
|  | DR median | **0.041** | **0.002** | **-** |  | 0.051 | 0.016 | **-** |

**Table S4**  Relationships between diversification rates and climate for the 483 genera that occur predominantly in China (>60% of species).

| Independent variable | Dependent variable | Genus level | |  |
| --- | --- | --- | --- | --- |
|  |  | *P* | *r*^2^ |  |
| MAP | MS: China (ε=0) | 0.284 | 0.002 | **+** |
|  | MS: China (ε=0.5) | 0.260 | 0.003 | **+** |
|  | MS: China (ε=0.9) | 0.202 | 0.003 | **+** |
|  | DR.median | 0.728 | <0.001 | **+** |
|  | MS: Global (ε=0) | 0.270 | 0.003 | **+** |
|  | MS: Global (ε=0.5) | 0.226 | 0.003 | **+** |
|  | MS: Global (ε=0.9) | 0.155 | 0.004 | **+** |
| MAT | MS: China (ε=0) | 0.435 | 0.001 | **+** |
|  | MS: China (ε=0.5) | 0.401 | 0.001 | **+** |
|  | MS: China (ε=0.9) | 0.292 | 0.002 | **+** |
|  | DR.median | 0.679 | <0.001 | **+** |
|  | MS: Global (ε=0) | 0.344 | 0.002 | **+** |
|  | MS: Global (ε=0.5) | 0.293 | 0.002 | **+** |
|  | MS: Global (ε=0.9) | 0.198 | 0.003 | **+** |

**Table S5**  Relationships between colonization times and species richness. Results are based on the genus-level tree (consensus and five selected trees) and using the mvBM model and estimated lambda (LA) model. Significant results (*P*<0.05) are boldfaced. Negative and positive relationships between richness and diversification rates are indicated by - and +.

|  |  |  | Regional richness | |  | Local richness | |  |
| --- | --- | --- | --- | --- | --- | --- | --- | --- |
| Models | Climate zone | Trees | *P* | *r*^2^ |  | *P* | *r*^2^ |  |
| mvBM | MAP | Consensus | **<0.001** | **0.755** |  | **<0.001** | **0.775** | + |
|  |  | 200th | **< 0.001** | **0.745** |  | **< 0.001** | **0.765** | + |
|  |  | 400th | **< 0.001** | **0.777** |  | **< 0.001** | **0.766** | + |
|  |  | 600th | **< 0.001** | **0.736** |  | **< 0.001** | **0.772** | + |
|  |  | 800th | **< 0.001** | **0.696** |  | **< 0.001** | **0.700** | + |
|  |  | 1000th | **< 0.001** | **0.728** |  | **< 0.001** | **0.735** | + |
|  | MAT | Consensus | **0.007** | **0.498** |  | **0.009** | **0.473** | + |
|  |  | 200th | **0.005** | **0.519** |  | **0.004** | **0.540** | + |
|  |  | 400th | **0.007** | **0.496** |  | **0.008** | **0.488** | + |
|  |  | 600th | **0.008** | **0.488** |  | **0.010** | **0.463** | + |
|  |  | 800th | **0.007** | **0.497** |  | **0.010** | **0.470** | + |
|  |  | 1000th | **0.007** | **0.504** |  | **0.006** | **0.506** | + |
| LA | MAP | Consensus | **< 0.001** | **0.758** | + | **< 0.001** | **0.789** | + |
|  |  | 200th | **< 0.001** | **0.777** | + | **< 0.001** | **0.786** | + |
|  |  | 400th | **< 0.001** | **0.766** | + | **< 0.001** | **0.785** | + |
|  |  | 600th | **< 0.001** | **0.772** | + | **< 0.001** | **0.766** | + |
|  |  | 800th | **< 0.001** | **0.652** | + | **< 0.001** | **0.736** | + |
|  |  | 1000th | **<0.001** | **0.652** | + | **<0.001** | **0.736** | + |
|  | MAT | Consensus | **0.010** | **0.464** | + | **0.016** | **0.424** | + |
|  |  | 200th | **0.011** | **0.459** | + | **0.016** | **0.423** | + |
|  |  | 400th | **0.011** | **0.457** | + | **0.018** | **0.414** | + |
|  |  | 600th | **0.011** | **0.456** | + | **0.016** | **0.421** | + |
|  |  | 800th | **0.010** | **0.464** | + | **0.016** | **0.424** | + |
|  |  | 1000th | **0.010** | **0.466** | + | **0.016** | **0.423** | + |

**Table S6**  Relationships between diversification rates and species richness of climatic zones (for precipitation) for the 47 most-species rich angiosperm families in China. Families are ordered based on their species richness (“Richness”). Diversification rates are estimated for genera based on the MS estimator, using ε=0.5 and species richness of genera in China only. Significant results (*P*<0.05) are boldfaced. Note that 3 families that are among the 50 most species-rich could not be included in these analyses because they have only one genus each. These are indicated with “NA”.

| Families | Richness | Local | | |  | | Regional | | | |  |
| --- | --- | --- | --- | --- | --- | --- | --- | --- | --- | --- | --- |
|  |  | *P* | *r* | *r*^2^ | |  | | *P* | *r* | *r*^2^ | |
| Compositae | 2091 | 0.298 | -0.268 | 0.072 | |  | | **0.004** | **0.662** | **0.439** | |
| Poaceae | 1590 | 0.052 | 0.479 | 0.229 | |  | | **0.001** | **0.714** | **0.510** | |
| Leguminosae | 1377 | **<0.001** | **-0.936** | **0.876** | |  | | **0.027** | **-0.533** | **0.285** | |
| Orchidaceae | 1244 | **<0.001** | **0.881** | **0.777** | |  | | **0.003** | **0.677** | **0.458** | |
| Lamiaceae | 920 | **<0.001** | **-0.928** | **0.861** | |  | | **0.001** | **-0.730** | **0.533** | |
| Rosaceae | 918 | **<0.001** | **0.847** | **0.717** | |  | | **<0.001** | **0.834** | **0.696** | |
| Ranunculaceae | 914 | 0.242 | 0.300 | 0.090 | |  | | 0.107 | -0.404 | 0.164 | |
| Cyperaceae | 835 | **0.005** | **-0.652** | **0.425** | |  | | 0.156 | -0.360 | 0.129 | |
| Ericaceae | 804 | **0.002** | **0.698** | **0.487** | |  | | **<0.001** | **0.820** | **0.672** | |
| Rubiaceae | 655 | **<0.001** | **-0.972** | **0.945** | |  | | **<0.001** | **-0.948** | **0.899** | |
| Primulaceae | 651 | **<0.001** | **0.832** | **0.693** | |  | | **0.001** | **0.740** | **0.548** | |
| Apiaceae | 545 | 0.400 | 0.218 | 0.048 | |  | | 0.203 | -0.325 | 0.105 | |
| Gesneriaceae | 469 | **0.005** | **0.664** | **0.441** | |  | | 0.481 | 0.190 | 0.036 | |
| Lauraceae | 437 | **0.007** | **0.643** | **0.413** | |  | | 0.576 | 0.151 | 0.023 | |
| Orobanchaceae | 429 | **0.048** | **0.486** | **0.236** | |  | | **<0.001** | **0.902** | **0.813** | |
| Papaveraceae | 418 | **0.040** | **0.501** | **0.251** | |  | | **<0.001** | **0.888** | **0.789** | |
| Gentianaceae | 416 | 0.101 | 0.411 | 0.169 | |  | | **0.002** | **0.692** | **0.479** | |
| Caryophyllaceae | 378 | 0.762 | -0.079 | 0.006 | |  | | **0.009** | **-0.613** | **0.376** | |
| Urticaceae | 378 | **<0.001** | **0.975** | **0.951** | |  | | **<0.001** | **0.908** | **0.825** | |
| Salicaceae | 363 | 0.207 | 0.322 | 0.104 | |  | | **0.003** | **0.668** | **0.446** | |
| Brassicaceae | 355 | **0.005** | **-0.644** | **0.414** | |  | | **0.003** | **-0.681** | **0.464** | |
| Apocynaceae | 353 | 0.098 | -0.414 | 0.172 | |  | | **<0.001** | **-0.971** | **0.943** | |
| Fagaceae | 299 | **0.008** | **0.620** | **0.384** | |  | | **<0.001** | **0.878** | **0.771** | |
| Berberidaceae | 298 | 0.520 | -0.168 | 0.028 | |  | | 0.260 | -0.289 | 0.084 | |
| Saxifragaceae | 290 | 0.743 | 0.086 | 0.007 | |  | | **0.040** | **0.503** | **0.253** | |
| Boraginaceae | 284 | **0.010** | **0.605** | **0.366** | |  | | 0.719 | -0.094 | 0.009 | |
| Acanthaceae | 269 | **<0.001** | **-0.933** | **0.871** | |  | | **<0.001** | **-0.897** | **0.805** | |
| Asparagaceae | 260 | **<0.001** | **0.842** | **0.709** | |  | | **<0.001** | **0.814** | **0.663** | |
| Celastraceae | 255 | **<0.001** | **0.812** | **0.659** | |  | | **0.001** | **0.740** | **0.547** | |
| Polygonaceae | 241 | **<0.001** | **-0.939** | **0.883** | |  | | **0.028** | **0.533** | **0.284** | |
| Balsaminaceae | 240 | NA | NA | NA | |  | | NA | NA | NA | |
| Crassulaceae | 231 | 0.949 | 0.017 | 0.000 | |  | | **0.014** | **0.584** | **0.341** | |
| Euphorbiaceae | 218 | 0.000 | 0.958 | 0.917 | |  | | 0.489 | 0.180 | 0.032 | |
| Malvaceae | 215 | 0.055 | 0.473 | 0.224 | |  | | **0.002** | **0.696** | **0.485** | |
| Zingiberaceae | 210 | **0.026** | **0.571** | **0.326** | |  | | **<0.001** | **0.838** | **0.703** | |
| Aquifoliaceae | 204 | NA | NA | NA | |  | | NA | NA | NA | |
| Amaranthaceae | 200 | **<0.001** | **0.945** | **0.894** | |  | | **<0.001** | **0.980** | **0.960** | |
| Araliaceae | 184 | **<0.001** | **-0.853** | **0.728** | |  | | 0.083 | -0.433 | 0.187 | |
| Begoniaceae | 173 | NA | NA | NA | |  | | NA | NA | NA | |
| Araceae | 162 | 0.165 | -0.353 | 0.124 | |  | | **0.044** | **-0.493** | **0.243** | |
| Vitaceae | 155 | 0.192 | 0.333 | 0.111 | |  | | **0.008** | **0.618** | **0.382** | |
| Oleaceae | 154 | **<0.001** | **0.879** | **0.773** | |  | | **<0.001** | **0.891** | **0.795** | |
| Campanulaceae | 153 | **0.022** | **-0.551** | **0.303** | |  | | 0.179 | -0.342 | 0.117 | |
| Plantaginaceae | 153 | 0.381 | -0.227 | 0.052 | |  | | 0.649 | 0.119 | 0.014 | |
| Sapindaceae | 147 | **0.035** | **0.514** | **0.264** | |  | | **0.012** | **-0.594** | **0.353** | |
| Theaceae | 146 | 0.057 | -0.520 | 0.270 | |  | | **0.009** | **0.669** | **0.448** | |
| Liliaceae | 142 | **0.015** | **0.576** | **0.332** | |  | | **0.016** | **0.576** | **0.331** | |
| Caprifoliaceae | 139 | **0.029** | **-0.528** | **0.279** | |  | | **<0.001** | **-0.830** | **0.689** | |
| Amaryllidaceae | 138 | **0.001** | **0.737** | **0.543** | |  | | **<0.001** | **0.910** | **0.829** | |
| Moraceae | 138 | **<0.001** | **0.978** | **0.956** | |  | | **0.003** | **0.670** | **0.449** | |

**Table S7** Relationships between diversification rates and species richness of climatic zones (for temperature) for the 47 most-species rich angiosperm families in China. Diversification rates are estimated for genera based on the MS estimator, using ε=0.5 and species richness of genera in China only. Significant results (*P*<0.05) are boldfaced. Note that 3 families are among the 50 most species-rich, but could not be included in these analyses because they have only one genus each. These are indicated with “NA”.

| Families | Richness | Local | | |  | Regional | | |
| --- | --- | --- | --- | --- | --- | --- | --- | --- |
|  |  | *P* | *r* | *r*^2^ |  | *P* | *r* | *r*^2^ |
| Compositae | 2091 | 0.267 | -0.319 | 0.102 |  | 0.357 | 0.267 | 0.071 |
| Poaceae | 1590 | 0.612 | 0.149 | 0.022 |  | **0.001** | **0.797** | **0.635** |
| Leguminosae | 1377 | **<0.001** | **-0.909** | **0.826** |  | 0.167 | -0.391 | 0.153 |
| Orchidaceae | 1244 | **<0.001** | **0.890** | **0.792** |  | **0.005** | **0.699** | **0.489** |
| Lamiaceae | 920 | **<0.001** | **-0.792** | **0.628** |  | **0.001** | **-0.797** | **0.635** |
| Rosaceae | 918 | **<0.001** | **0.914** | **0.835** |  | **<0.001** | **0.961** | **0.924** |
| Ranunculaceae | 914 | 0.233 | 0.341 | 0.116 |  | 0.726 | -0.103 | 0.011 |
| Cyperaceae | 835 | **0.014** | **-0.638** | **0.408** |  | 0.064 | -0.508 | 0.258 |
| Ericaceae | 804 | 0.282 | 0.309 | 0.095 |  | **<0.001** | **0.889** | **0.790** |
| Rubiaceae | 655 | **<0.001** | **-0.878** | **0.772** |  | **<0.001** | **-0.894** | **0.799** |
| Primulaceae | 651 | **<0.001** | **0.961** | **0.923** |  | **<0.001** | **0.831** | **0.690** |
| Apiaceae | 545 | **0.013** | **0.642** | **0.412** |  | **0.046** | **-0.541** | **0.293** |
| Gesneriaceae | 469 | **0.021** | **0.631** | **0.398** |  | 0.674 | 0.129 | 0.017 |
| Lauraceae | 437 | 0.734 | -0.110 | 0.012 |  | 0.251 | -0.360 | 0.129 |
| Orobanchaceae | 429 | 0.327 | 0.283 | 0.080 |  | **<0.001** | **0.868** | **0.753** |
| Papaveraceae | 418 | 0.125 | -0.430 | 0.185 |  | **<0.001** | **0.820** | **0.672** |
| Gentianaceae | 416 | 0.082 | 0.481 | 0.231 |  | 0.139 | 0.416 | 0.173 |
| Caryophyllaceae | 378 | 0.219 | -0.351 | 0.123 |  | **0.002** | **-0.762** | **0.580** |
| Urticaceae | 378 | **<0.001** | 0.953 | 0.909 |  | **<0.001** | **0.891** | **0.793** |
| Salicaceae | 363 | 0.680 | 0.121 | 0.015 |  | 0.379 | 0.255 | 0.065 |
| Brassicaceae | 355 | **<0.001** | **-0.812** | **0.660** |  | **0.049** | **-0.535** | **0.286** |
| Apocynaceae | 353 | 0.239 | -0.337 | 0.113 |  | **<0.001** | **-0.939** | **0.881** |
| Fagaceae | 299 | 0.241 | 0.336 | 0.113 |  | **<0.001** | **0.845** | **0.714** |
| Berberidaceae | 298 | 0.321 | -0.286 | 0.082 |  | **0.001** | **-0.779** | **0.606** |
| Saxifragaceae | 290 | 0.482 | 0.205 | 0.042 |  | 0.607 | 0.151 | 0.023 |
| Boraginaceae | 284 | 0.659 | 0.130 | 0.017 |  | 0.773 | -0.085 | 0.007 |
| Acanthaceae | 269 | **<0.001** | **-0.929** | **0.863** |  | **0.001** | **-0.828** | **0.686** |
| Asparagaceae | 260 | **0.048** | **0.536** | **0.287** |  | **<0.001** | **0.933** | **0.871** |
| Celastraceae | 255 | **0.001** | **0.768** | **0.590** |  | **0.005** | **0.708** | **0.501** |
| Polygonaceae | 241 | **<0.001** | **-0.896** | **0.803** |  | 0.608 | -0.150 | 0.023 |
| Balsaminaceae | 240 | NA | NA | NA |  | NA | NA | NA |
| Crassulaceae | 231 | 0.566 | -0.168 | 0.028 |  | **0.040** | **0.553** | **0.306** |
| Euphorbiaceae | 218 | **<0.001** | **0.951** | **0.905** |  | 0.828 | 0.064 | 0.004 |
| Malvaceae | 215 | 0.079 | 0.484 | 0.235 |  | 0.096 | 0.462 | 0.213 |
| Zingiberaceae | 210 | 0.132 | 0.461 | 0.212 |  | **<0.001** | **0.903** | **0.815** |
| Aquifoliaceae | 204 | NA | NA | NA |  | NA | NA | NA |
| Amaranthaceae | 200 | **<0.001** | **0.950** | **0.903** |  | **<0.001** | **0.880** | **0.774** |
| Araliaceae | 184 | 0.941 | -0.022 | 0.000 |  | 0.134 | 0.421 | 0.177 |
| Begoniaceae | 173 | NA | NA | NA |  | NA | NA | NA |
| Araceae | 162 | **<0.001** | **-0.833** | **0.694** |  | **<0.001** | **-0.966** | **0.933** |
| Vitaceae | 155 | **<0.001** | **0.844** | **0.713** |  | **0.007** | **0.684** | **0.467** |
| Oleaceae | 154 | **<0.001** | **0.840** | **0.705** |  | **<0.001** | **0.857** | **0.735** |
| Campanulaceae | 153 | 0.210 | -0.357 | 0.128 |  | 0.197 | -0.366 | 0.134 |
| Plantaginaceae | 153 | 0.120 | -0.435 | 0.190 |  | 0.113 | 0.443 | 0.196 |
| Sapindaceae | 147 | 0.678 | -0.122 | 0.015 |  | **<0.001** | **-0.892** | **0.795** |
| Theaceae | 146 | 0.211 | -0.409 | 0.167 |  | 0.752 | 0.108 | 0.012 |
| Liliaceae | 142 | **0.029** | **0.581** | **0.338** |  | **0.044** | **0.546** | **0.298** |
| Caprifoliaceae | 139 | 0.877 | -0.046 | 0.002 |  | **0.041** | **-0.550** | **0.303** |
| Amaryllidaceae | 138 | **0.011** | **0.656** | **0.431** |  | **0.017** | **0.624** | **0.389** |
| Moraceae | 138 | **<0.001** | **0.917** | **0.842** |  | **0.005** | **0.699** | **0.488** |

**Table S8**  Relationships between timing of first colonization and species richness of climatic zones (for precipitation; MAP) for the 50 largest angiosperm families in China. Results are based on the species-level consensus tree and the mvBM model. Families are ordered based on their species richness. Significant results (*P*<0.05) are boldfaced.

| Family | Richness | Local | |  | Regional | |
| --- | --- | --- | --- | --- | --- | --- |
|  |  | *P* | *r*^2^ |  | *P* | *r*^2^ |
| Compositae | 2091 | **<0.001** | 0.654 |  | **0.001** | 0.515 |
| Poaceae | 1590 | **0.031** | 0.274 |  | 0.203 | 0.106 |
| Leguminosae | 1377 | **0.002** | 0.472 |  | 0.062 | 0.213 |
| Orchidaceae | 1244 | 0.053 | 0.242 |  | **0.022** | 0.320 |
| Lamiaceae | 920 | **0.002** | 0.494 |  | **0.001** | 0.550 |
| Rosaceae | 918 | **0.022** | 0.301 |  | **0.017** | 0.327 |
| Ranunculaceae | 914 | **<0.001** | 0.614 |  | **0.003** | 0.458 |
| Cyperaceae | 835 | **0.006** | 0.408 |  | 0.080 | 0.190 |
| Ericaceae | 804 | **0.001** | 0.555 |  | **0.002** | 0.527 |
| Rubiaceae | 655 | **0.020** | 0.313 |  | **0.009** | 0.378 |
| Primulaceae | 651 | **0.006** | 0.400 |  | **<0.001** | 0.585 |
| Apiaceae | 545 | **0.003** | 0.446 |  | **0.013** | 0.345 |
| Gesneriaceae | 469 | **0.010** | 0.464 |  | **0.004** | 0.551 |
| Lauraceae | 437 | 0.272 | 0.169 |  | 0.187 | 0.234 |
| Orobanchaceae | 429 | **0.036** | 0.261 |  | **0.004** | 0.428 |
| Papaveraceae | 418 | 0.055 | 0.224 |  | **0.041** | 0.251 |
| Gentianaceae | 416 | **0.007** | 0.415 |  | **0.006** | 0.423 |
| Caryophyllaceae | 378 | 0.237 | 0.098 |  | **0.032** | 0.288 |
| Urticaceae | 378 | **0.001** | 0.564 |  | **0.003** | 0.510 |
| Salicaceae | 363 | 0.568 | 0.022 |  | 0.377 | 0.052 |
| Brassicaceae | 355 | **<0.001** | 0.628 |  | **<0.001** | 0.772 |
| Apocynaceae | 353 | **0.004** | 0.491 |  | **0.005** | 0.467 |
| Fagaceae | 299 | 0.267 | 0.135 |  | 0.151 | 0.215 |
| Berberidaceae | 298 | **0.017** | 0.326 |  | **0.007** | 0.394 |
| Saxifragaceae | 290 | **<0.001** | 0.690 |  | **0.017** | 0.342 |
| Boraginaceae | 284 | 0.889 | 0.001 |  | 0.563 | 0.023 |
| Acanthaceae | 269 | **0.020** | 0.432 |  | **0.045** | 0.343 |
| Asparagaceae | 260 | 0.090 | 0.191 |  | 0.307 | 0.074 |
| Celastraceae | 255 | **0.044** | 0.259 |  | 0.077 | 0.206 |
| Polygonaceae | 241 | 0.060 | 0.216 |  | **0.011** | 0.356 |
| Balsaminaceae | 240 | 0.056 | 0.253 |  | **0.016** | 0.371 |
| Crassulaceae | 231 | **0.003** | 0.455 |  | **0.010** | 0.370 |
| Euphorbiaceae | 218 | **0.007** | 0.393 |  | **0.008** | 0.386 |
| Malvaceae | 215 | **0.002** | 0.554 |  | **0.003** | 0.530 |
| Zingiberaceae | 210 | **<0.001** | 0.686 |  | **<0.001** | 0.745 |
| Aquifoliaceae | 204 | 0.504 | 0.066 |  | 0.217 | 0.209 |
| Amaranthaceae | 200 | 0.933 | 0.000 |  | 0.492 | 0.032 |
| Araliaceae | 184 | 0.083 | 0.248 |  | 0.212 | 0.138 |
| Begoniaceae | 173 | 0.206 | 0.217 |  | 0.053 | 0.434 |
| Araceae | 162 | **0.021** | 0.398 |  | **0.022** | 0.394 |
| Vitaceae | 155 | 0.059 | 0.311 |  | 0.111 | 0.234 |
| Oleaceae | 154 | **0.025** | 0.352 |  | 0.051 | 0.281 |
| Campanulaceae | 153 | **0.007** | 0.415 |  | **0.049** | 0.250 |
| Plantaginaceae | 153 | **0.005** | 0.414 |  | 0.374 | 0.053 |
| Sapindaceae | 147 | 0.121 | 0.204 |  | 0.423 | 0.059 |
| Theaceae | 146 | 0.755 | 0.017 |  | 0.255 | 0.209 |
| Liliaceae | 142 | **0.014** | 0.382 |  | 0.087 | 0.208 |
| Caprifoliaceae | 139 | **0.004** | 0.451 |  | 0.075 | 0.209 |
| Amaryllidaceae | 138 | 0.670 | 0.012 |  | 0.545 | 0.025 |
| Moraceae | 138 | **0.036** | 0.370 |  | **0.050** | 0.331 |

**Table S9** Relationships between timing of first colonization and species richness of climatic zones (for temperature; MAT) for the 50 largest angiosperm families in China. Results are based on the species-level consensus tree and the mvBM model. Families are ordered based on their species richness. Significant results (*P*<0.05) are boldfaced.

| Family | Species | Local | |  | Regional | |
| --- | --- | --- | --- | --- | --- | --- |
|  |  | *P* | *r*^2^ |  | *P* | *r*^2^ |
| Compositae | 2091 | 0.869 | 0.003 |  | 0.174 | 0.161 |
| Poaceae | 1590 | **0.016** | 0.425 |  | 0.054 | 0.297 |
| Leguminosae | 1377 | **0.007** | 0.502 |  | 0.424 | 0.059 |
| Orchidaceae | 1244 | 0.241 | 0.149 |  | 0.143 | 0.223 |
| Lamiaceae | 920 | **0.002** | 0.615 |  | **0.003** | 0.569 |
| Rosaceae | 918 | 0.728 | 0.011 |  | 0.442 | 0.055 |
| Ranunculaceae | 914 | 0.735 | 0.011 |  | 0.062 | 0.282 |
| Cyperaceae | 835 | **0.001** | 0.632 |  | 0.103 | 0.223 |
| Ericaceae | 804 | 0.955 | 0.000 |  | 0.619 | 0.026 |
| Rubiaceae | 655 | 0.935 | 0.001 |  | 0.955 | 0.000 |
| Primulaceae | 651 | **0.047** | 0.312 |  | **0.016** | 0.421 |
| Apiaceae | 545 | 0.868 | 0.003 |  | 0.181 | 0.171 |
| Gesneriaceae | 469 | 0.400 | 0.080 |  | 0.180 | 0.190 |
| Lauraceae | 437 | 0.183 | 0.274 |  | 0.116 | 0.359 |
| Orobanchaceae | 429 | 0.667 | 0.019 |  | 0.232 | 0.140 |
| Papaveraceae | 418 | 0.350 | 0.080 |  | 0.245 | 0.120 |
| Gentianaceae | 416 | 0.383 | 0.077 |  | 0.126 | 0.218 |
| Caryophyllaceae | 378 | 0.724 | 0.012 |  | 0.092 | 0.236 |
| Urticaceae | 378 | 0.304 | 0.117 |  | 0.346 | 0.099 |
| Salicaceae | 363 | 0.867 | 0.003 |  | 0.674 | 0.017 |
| Brassicaceae | 355 | **0.044** | 0.320 |  | **0.004** | 0.537 |
| Apocynaceae | 353 | **<0.001** | 0.940 |  | **0.001** | 0.756 |
| Fagaceae | 299 | 0.109 | 0.260 |  | 0.093 | 0.281 |
| Berberidaceae | 298 | 0.064 | 0.302 |  | 0.299 | 0.107 |
| Saxifragaceae | 290 | 0.262 | 0.113 |  | 0.137 | 0.190 |
| Boraginaceae | 284 | 0.575 | 0.029 |  | 0.669 | 0.017 |
| Acanthaceae | 269 | **<0.001** | 0.843 |  | **0.001** | 0.834 |
| Asparagaceae | 260 | 0.384 | 0.085 |  | 0.571 | 0.037 |
| Celastraceae | 255 | 0.051 | 0.330 |  | 0.055 | 0.321 |
| Polygonaceae | 241 | 0.650 | 0.021 |  | 0.178 | 0.174 |
| Balsaminaceae | 240 | 0.476 | 0.058 |  | 0.506 | 0.051 |
| Crassulaceae | 231 | 0.419 | 0.066 |  | 0.375 | 0.079 |
| Euphorbiaceae | 218 | **<0.001** | 0.823 |  | **<0.001** | 0.741 |
| Malvaceae | 215 | **<0.001** | 0.944 |  | **<0.001** | 0.893 |
| Zingiberaceae | 210 | 0.660 | 0.020 |  | 0.103 | 0.243 |
| Aquifoliaceae | 204 | 0.170 | 0.288 |  | 0.179 | 0.278 |
| Amaranthaceae | 200 | **0.046** | 0.340 |  | 0.444 | 0.060 |
| Araliaceae | 184 | 0.349 | 0.098 |  | 0.201 | 0.175 |
| Begoniaceae | 173 | 0.013 | 0.669 |  | <0.001 | 0.892 |
| Araceae | 162 | 0.175 | 0.194 |  | 0.159 | 0.208 |
| Vitaceae | 155 | 0.122 | 0.306 |  | 0.092 | 0.353 |
| Oleaceae | 154 | **0.028** | 0.472 |  | 0.052 | 0.393 |
| Campanulaceae | 153 | 0.298 | 0.108 |  | 0.214 | 0.150 |
| Plantaginaceae | 153 | 0.706 | 0.015 |  | 0.906 | 0.001 |
| Sapindaceae | 147 | **0.043** | 0.419 |  | **0.004** | 0.670 |
| Theaceae | 146 | 0.098 | 0.654 |  | 0.141 | 0.568 |
| Liliaceae | 142 | 0.483 | 0.056 |  | 0.298 | 0.119 |
| Caprifoliaceae | 139 | 0.954 | 0.000 |  | 0.070 | 0.291 |
| Amaryllidaceae | 138 | 0.886 | 0.002 |  | 0.656 | 0.021 |
| Moraceae | 138 | 0.555 | 0.061 |  | 0.268 | 0.199 |

**Table S10** Number of families in which species richness patterns were significantly related to colonization time, diversification rate, both, or neither, with colonization times estimated using the estimated lambda model. We present results for relationships between richness of climatic bins (local and regional) and diversification and colonization time for the 47 most-species rich families in China (including 80% of the species in our phylogenetic tree). These are followed by results for the 10 most species-rich families (including 42% of the species in our tree). Climatic variables are MAP (mean annual precipitation) and MAT (mean annual temperature). Diversification rates were estimated based on stem-group ages of genera with ε=0.5, including only species in China.

|  |  | MAP | |  | MAT | |
| --- | --- | --- | --- | --- | --- | --- |
|  |  | Local | Regional |  | Local | Regional |
| 47 families | Time not rate | 20 | 20 |  | 3 | 5 |
|  | Rate not time | 6 | 9 |  | 12 | 15 |
|  | Time and rate | 15 | 17 |  | 3 | 4 |
|  | Not time or rate | 6 | 1 |  | 29 | 23 |
|  |  |  |  |  |  |  |
| 10 families | Time not rate | 7 | 5 |  | 0 | 2 |
|  | Rate not time | 0 | 1 |  | 1 | 2 |
|  | Time and rate | 3 | 4 |  | 1 | 2 |
|  | Not time or rate | 0 | 0 |  | 8 | 4 |

**Table S11** Phylogenetic signal in climatic variables for the 50 largest angiosperm families in China. Estimated values of phylogenetic signal (λ) are shown, along with *P*-values testing whether estimated signal is significantly different from zero (*P*_λ=0_). Significant results (*P*<0.05) are boldfaced.

| Family | Species | MAP | |  | MAT | |
| --- | --- | --- | --- | --- | --- | --- |
|  |  |  |  |  |  |  |
|  |  | λ | *P*_λ=0_ |  | λ | *P*_λ=0_ |
| Compositae | 2091 | 0.570 | **<0.001** |  | 0.591 | **<0.001** |
| Poaceae | 1590 | 0.873 | **<0.001** |  | 0.852 | **<0.001** |
| Leguminosae | 1377 | 0.582 | **<0.001** |  | 0.625 | **<0.001** |
| Orchidaceae | 1244 | 0.444 | **<0.001** |  | 0.503 | **<0.001** |
| Lamiaceae | 920 | 0.630 | **<0.001** |  | 0.660 | **<0.001** |
| Rosaceae | 918 | 0.469 | **<0.001** |  | 0.655 | **<0.001** |
| Ranunculaceae | 914 | 0.187 | **<0.001** |  | 0.238 | **<0.001** |
| Cyperaceae | 835 | 0.362 | **<0.001** |  | 0.394 | **<0.001** |
| Ericaceae | 804 | 0.212 | **<0.001** |  | 0.353 | **<0.001** |
| Rubiaceae | 655 | 0.473 | **<0.001** |  | 0.662 | **<0.001** |
| Primulaceae | 651 | 0.403 | **<0.001** |  | 0.459 | **<0.001** |
| Apiaceae | 545 | 0.646 | **<0.001** |  | 0.491 | **<0.001** |
| Gesneriaceae | 469 | 0.454 | **<0.001** |  | 0.364 | **<0.001** |
| Lauraceae | 437 | 0.323 | 1.000 |  | 0.119 | **<0.001** |
| Orobanchaceae | 429 | 0.302 | **<0.001** |  | 0.319 | **<0.001** |
| Papaveraceae | 418 | 0.166 | 1.000 |  | <0.001 | 1.000 |
| Gentianaceae | 416 | 0.277 | **<0.001** |  | 0.347 | **<0.001** |
| Caryophyllaceae | 378 | 0.022 | 0.585 |  | 0.204 | **0.001** |
| Urticaceae | 378 | 0.276 | **<0.001** |  | 0.321 | **<0.001** |
| Salicaceae | 363 | 0.348 | **<0.001** |  | 0.473 | **<0.001** |
| Brassicaceae | 355 | 0.403 | **<0.001** |  | 0.373 | **<0.001** |
| Apocynaceae | 353 | 0.478 | **<0.001** |  | 0.586 | **<0.001** |
| Fagaceae | 299 | 0.278 | **<0.001** |  | 0.396 | **<0.001** |
| Berberidaceae | 298 | 0.536 | **<0.001** |  | 0.694 | **<0.001** |
| Saxifragaceae | 290 | 0.223 | **<0.001** |  | 0.216 | **<0.001** |
| Boraginaceae | 284 | 0.570 | **<0.001** |  | 0.522 | **<0.001** |
| Acanthaceae | 269 | 0.229 | 0.089 |  | 0.133 | **<0.001** |
| Asparagaceae | 260 | 0.742 | **<0.001** |  | 0.799 | **<0.001** |
| Celastraceae | 255 | 0.233 | **<0.001** |  | 0.379 | **<0.001** |
| Polygonaceae | 241 | 0.328 | **<0.001** |  | 0.184 | **<0.001** |
| Balsaminaceae | 240 | 0.241 | **0.001** |  | 0.384 | **<0.001** |
| Crassulaceae | 231 | 0.537 | **<0.001** |  | 0.390 | **<0.001** |
| Euphorbiaceae | 218 | 0.350 | **<0.001** |  | 0.538 | **<0.001** |
| Malvaceae | 215 | 0.413 | **<0.001** |  | 0.566 | **<0.001** |
| Zingiberaceae | 210 | 0.395 | **<0.001** |  | 0.488 | **<0.001** |
| Aquifoliaceae | 204 | 0.000 | 1.000 |  | 0.626 | 0.255 |
| Amaranthaceae | 200 | 0.607 | **<0.001** |  | 0.470 | **<0.001** |
| Araliaceae | 184 | 0.806 | **0.001** |  | 0.771 | **0.006** |
| Begoniaceae | 173 | 0.021 | 0.655 |  | 0.005 | 0.948 |
| Araceae | 162 | 0.363 | **<0.001** |  | 0.434 | **<0.001** |
| Vitaceae | 155 | 0.185 | 0.102 |  | 0.318 | **0.001** |
| Oleaceae | 154 | 0.618 | **<0.001** |  | 0.665 | **<0.001** |
| Campanulaceae | 153 | 0.259 | **<0.001** |  | 0.334 | **<0.001** |
| Plantaginaceae | 153 | 0.479 | **<0.001** |  | 0.661 | **<0.001** |
| Sapindaceae | 147 | 0.160 | **<0.001** |  | 0.306 | **<0.001** |
| Theaceae | 146 | 0.000 | 1.000 |  | 0.251 | 0.257 |
| Liliaceae | 142 | 0.686 | **<0.001** |  | 0.681 | **<0.001** |
| Caprifoliaceae | 139 | 0.000 | 1.000 |  | 0.261 | **0.004** |
| Amaryllidaceae | 138 | 0.445 | **<0.001** |  | 0.596 | **<0.001** |
| Moraceae | 138 | 0.139 | **0.014** |  | 0.498 | **<0.001** |

**
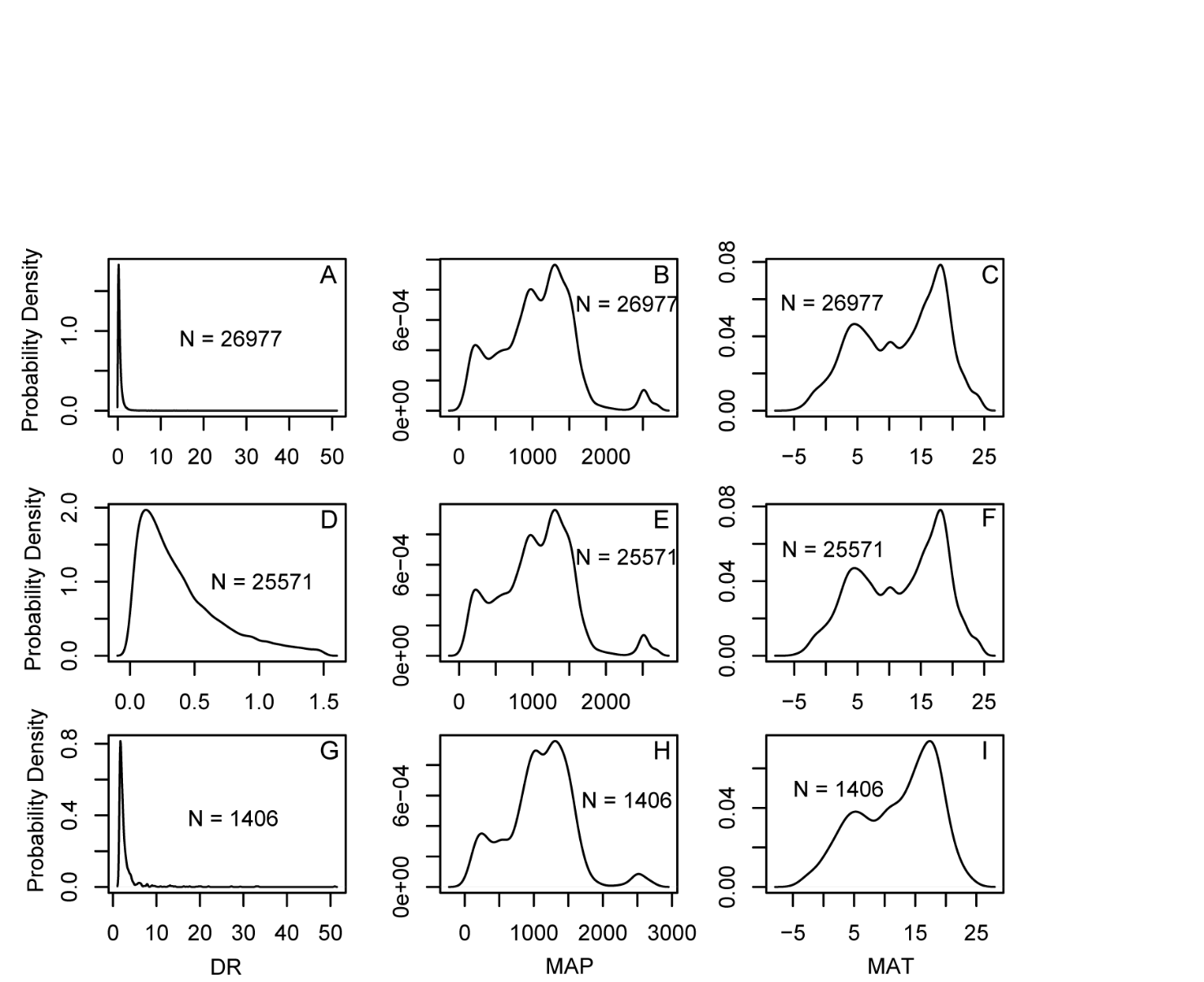
**

**Fig. S1** Distribution of DR values among species and climatic zones. Graphs A–C show the probability density distribution of species-level diversification rates (DR) across all species (A) and for different species-level values of MAP and MAT (B, C). Graphs (D–F) show distributions for species with DR <1.5 (D, E, F). Species with DR >1.5 are shown in graphs G–I.


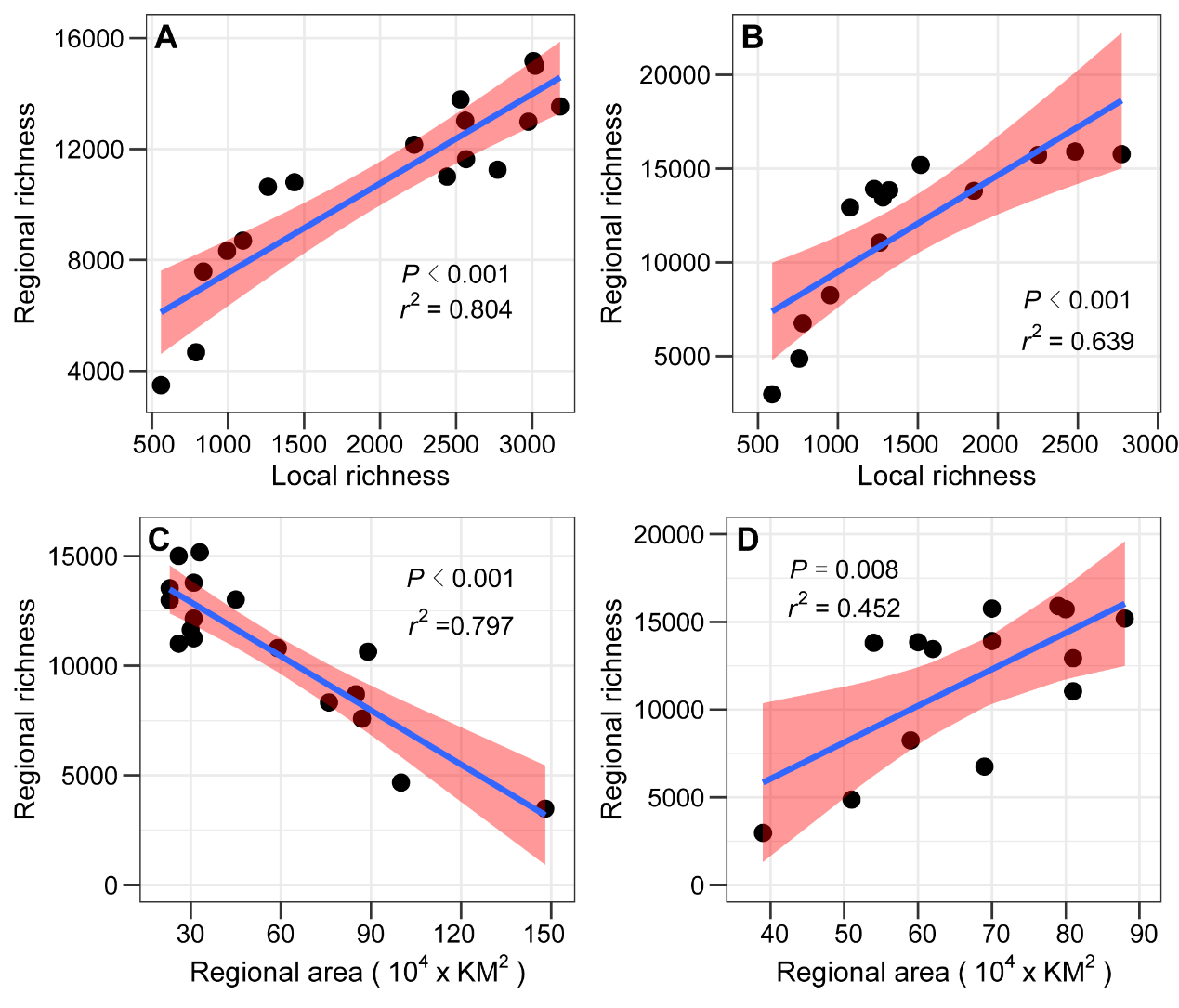


**Fig. S2**  Relationships between local and regional richness and between regional richness and area for different climatic bins. A and C are results for mean annual precipitation, and B and D are for mean annual temperature. Area reflects the number of 100x100 km grid cells that are in a given climatic bin. The pink color indicates the 95% confidence interval.


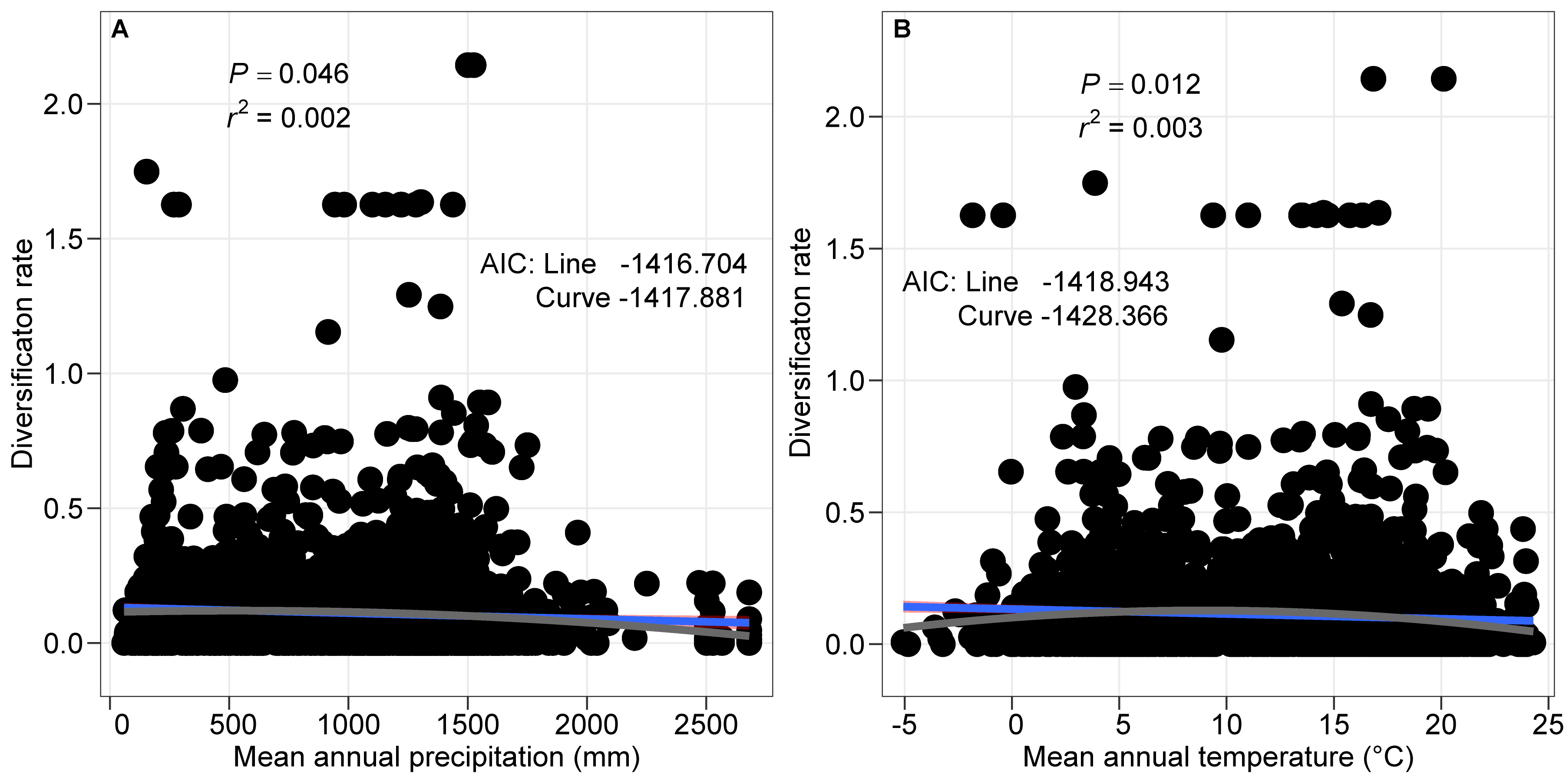


**Fig. S3**  Relationships between diversification rates and mean temperature and precipitation among genera. Climate was based on mean values among species in each genus, with values for each species based on mean values among grid cells in which they occur (for species and grid cells occurring in China). Diversification rates were estimated using the MS estimator with stem-group ages of genera with ε=0.5, including only species in China. Use of alternative values of ε (0, 0.9) and global richness of genera yielded similar results (Table S3). The linear and nonlinear relationships showed in blue and gray respectively. Nonlinear models were significantly better than that of linear models for both MAP (*P*=0.005) and MAT (*P* =0.002).


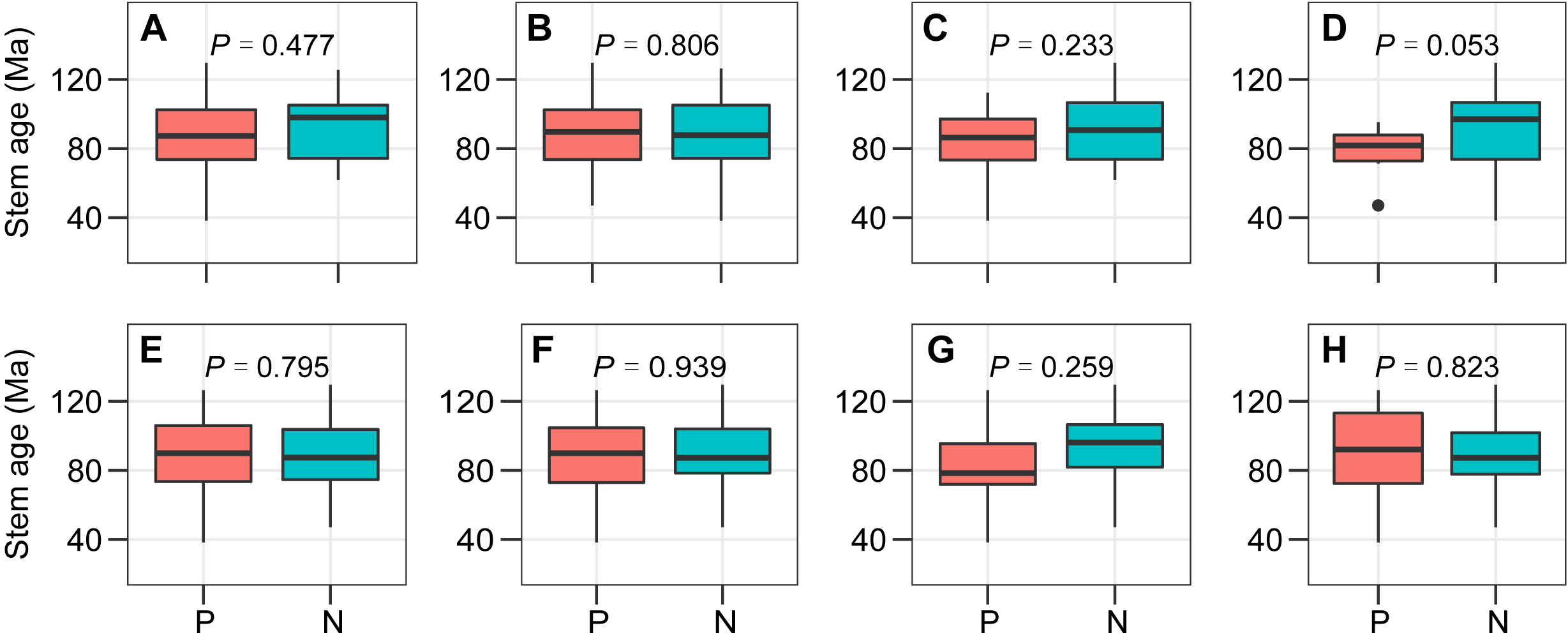


**Fig. S4**  Comparison of stem ages of families that support the time hypothesis relative to those that do not (A, B, C, D) and those that support the diversification-rate hypothesis or not (E, F, G, H). Only the 47 most species-rich families are included. The orange boxes (P) represent significant, positive relationship between species richness and time or diversification rate. The blue boxes (N) represent negative or no significant relationships between species richness and time or diversification rate. The black horizontal line is the median stem age; upper and lower limits of the box indicate 95% confidence interval. *P*-values are based on a t-test. (A) Local richness and first colonization time for MAP; (B) regional richness and first colonization time for MAP; (C) local richness and first colonization time for MAT; (D) regional richness and first colonization time for MAT; (E) local richness and diversification rate for MAP; (F) regional richness and diversification rate for MAP; (G) local richness and diversification rate for MAT; (H) regional richness and diversification rate for MAT.


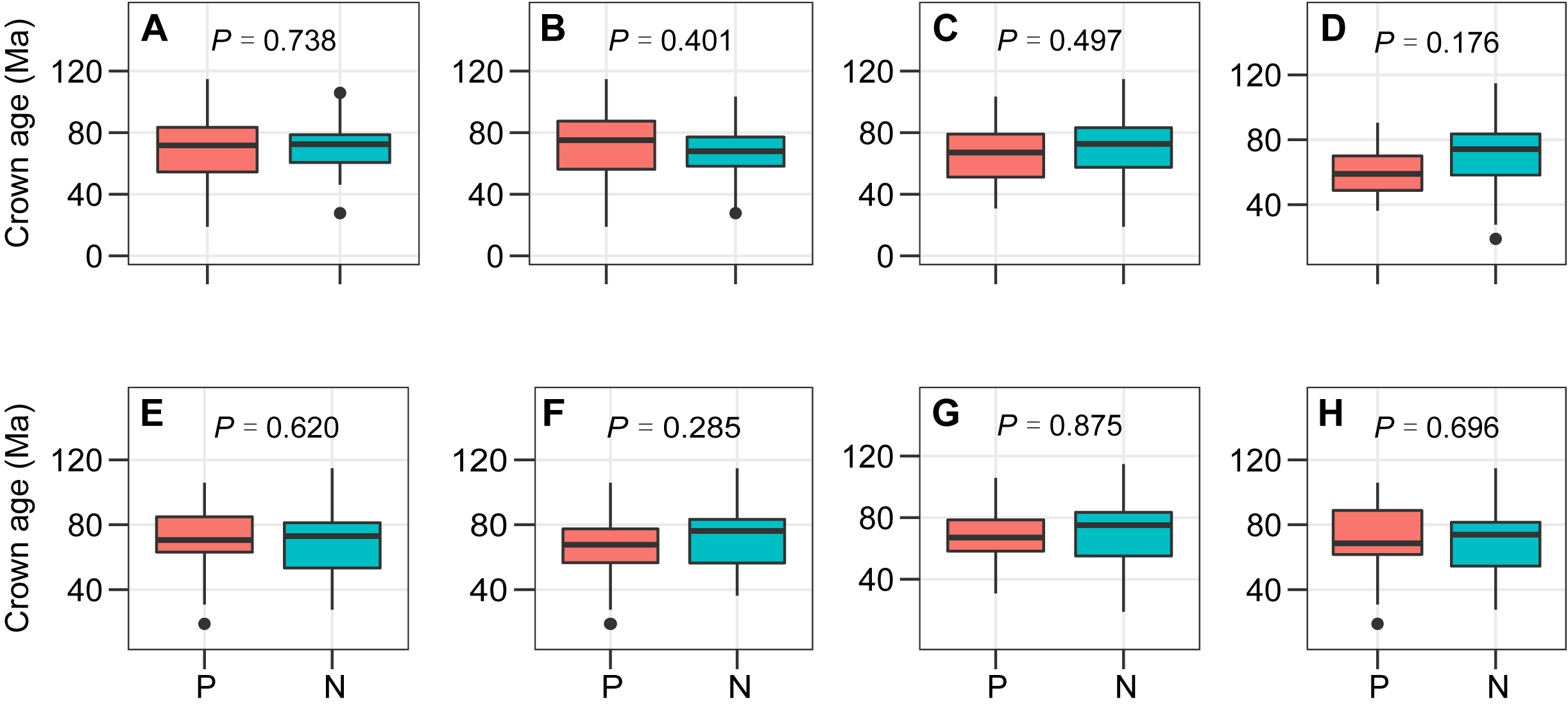


**Fig. S5**  Comparison of crown-group ages of families that support the time hypothesis relative to those that do not (A, B, C, D) and those that support the diversification-rate hypothesis or not (E, F, G, H). Only the 47 most species-rich families are included. The orange boxes (P) represent significant, positive relationship between species richness and time or diversification rate. The blue boxes (N) represent negative or no significant relationships between species richness and time or diversification rate. The black horizontal line is the median stem age; upper and lower limits of the box indicate 95% confidence interval. *P*-values are based on a t-test. (A) Local richness and first colonization time for MAP; (B) regional richness and first colonization time for MAP; (C) local richness and first colonization time for MAT; (D) regional richness and first colonization time for MAT; (E) local richness and diversification rate for MAP; (F) regional richness and diversification rate for MAP; (G) local richness and diversification rate for MAT; (H) regional richness and diversification rate for MAT.


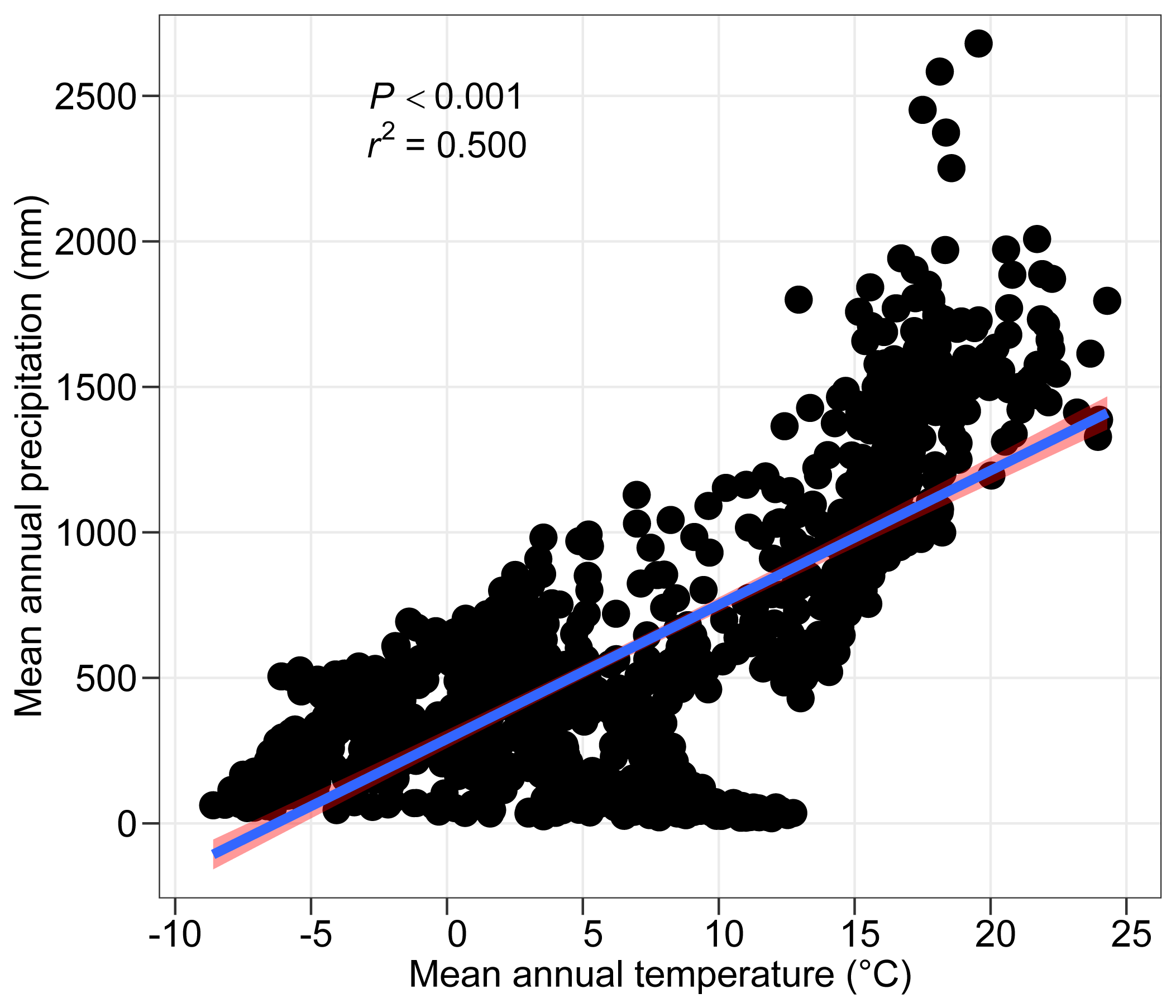


**Fig. S6** Relationship between mean annual temperature (MAT) and mean annual precipitation (MAP) among 943 grid cells in China.
